# Substrate spectrum of PPM1D in the cellular response to DNA double-strand breaks

**DOI:** 10.1016/j.isci.2022.104892

**Published:** 2022-08-09

**Authors:** Justus F. Gräf, Ivan Mikicic, Xiaofei Ping, Claudia Scalera, Katharina Mayr, Lukas S. Stelzl, Petra Beli, Sebastian A. Wagner

**Affiliations:** 1 Institute of Molecular Biology (IMB), 55128 Mainz, Germany; 2 Faculty of Biology, Johannes Gutenberg University, 55128 Mainz, Germany; 3 KOMET 1, Institute of Physics, Johannes Gutenberg University, 55099 Mainz, Germany; 4 Institute of Developmental Biology and Neurobiology (IDN), Johannes Gutenberg University, 55128 Mainz, Germany; 5 Department of Medicine, Hematology/Oncology, Goethe University, 60590 Frankfurt, Germany; 6 German Cancer Consortium (DKTK) and German Cancer Research Center (DKFZ), 69120 Heidelberg, Germany; 7 Frankfurt Cancer Institute (FCI), 60596 Frankfurt, Germany

**Keywords:** Biochemistry, molecular biology, cancer, proteomics

## Abstract

PPM1D is a p53-regulated protein phosphatase that modulates the DNA damage response (DDR) and is frequently altered in cancer. Here, we employed chemical inhibition of PPM1D and quantitative mass spectrometry-based phosphoproteomics to identify the substrates of PPM1D upon induction of DNA double-strand breaks (DSBs) by etoposide. We identified 73 putative PPM1D substrates that are involved in DNA repair, regulation of transcription, and RNA processing. One-third of DSB-induced S/TQ phosphorylation sites are dephosphorylated by PPM1D, demonstrating that PPM1D only partially counteracts ATM/ATR/DNA-PK signaling. PPM1D-targeted phosphorylation sites are found in a specific amino acid sequence motif that is characterized by glutamic acid residues, high intrinsic disorder, and poor evolutionary conservation. We identified a functionally uncharacterized protein Kanadaptin as ATM and PPM1D substrate upon DSB induction. We propose that PPM1D plays a role during the response to DSBs by regulating the phosphorylation of DNA- and RNA-binding proteins in intrinsically disordered regions.

## Highlights

• MS-based phosphoproteomic profiling of PPM1D substrates in U2OS and HCT116 cells
• PPM1D counteracts ATM in the cellular response to DNA double-strand breaks
• PPM1D target sites localize to glutamic acid-rich regions with high intrinsic disorder
• Kanadaptin is a putative DNA damage response factor regulated by ATM and PPM1D

## Graphical abstract

**Figure:**
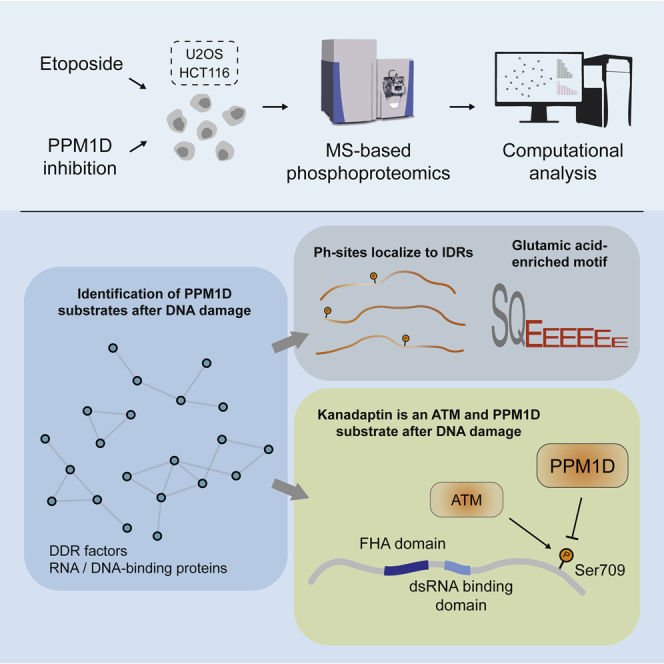

## Introduction

The DNA damage response (DDR) relies on the activity of Phosphatidylinositol 3-kinase-related kinases (PIKKs) ATM, ATR, and DNA-PK (Blackford and Jackson, 2017). These kinases are activated to a different extent based on the type of DNA damage and often act redundantly to regulate single- and double-strand DNA break (DSB) processing, replication fork stability, the cell-cycle arrest, and apoptosis (Yan et al., 2014; Lanz et al., 2019). Multiple phosphatases counteract phosphorylation by PIKKs to prevent untimely activation and to cease the DDR (Peng and Maller, 2010). One of them is PPM1D (also known as WIP1, Wild-type p53-induced protein 1), which is activated by p53 and forms a negative feedback loop with the p53 signaling axis. PPM1D exerts its suppressive function on p53 through multiple mechanisms, including the dephosphorylation of p53 and multiple DDR effector proteins (Lu et al., 2005; Yamaguchi et al., 2007). PPM1D dephosphorylates ATM at S1981, histone variant H2AX at S139 (γH2AX), CHEK1 at S345, and p53 at S15 (Lu et al., 2005; Shreeram et al., 2006; Cha et al., 2010). Ppm1d deficiency in mice is able to partially rescue ATM-deficiency phenotypes, including reduced γH2AX and p21 levels and chromosomal instability (Darlington et al., 2012).

Tumors with wild-type p53 often rely on other mechanisms for inactivating the p53 tumor suppressor function (Pecháčková et al., 2017). Amplification of the *PPM1D* gene or gain-of-function mutations are commonly found in solid cancer, in particular in tumors with wild-type p53 (Li et al., 2002; Natrajan et al., 2009). Additionally, pathogenic variants in *PPM1D* are frequently identified in patients with clonal hematopoiesis of indeterminate potential (CHIP) that underwent treatment with DNA damaging agents (Bolton et al., 2020). The pathogenic variants in PPM1D observed in subjects with CHIP are mainly truncating mutations in exon 6 of *PPM1D*. These truncations lead to the expression of PPM1D variants that show elevated protein levels and phosphatase activity (Kahn et al., 2018). Notably, one-fifth of patients with therapy-related MDS or AML harbor mutations in *PPM1D* (Hsu et al., 2018). In addition, hematopoietic stem cells with hyperactive PPM1D outcompete others after treatment with DNA-damaging agents etoposide or doxorubicin (Hsu et al., 2018). In mouse models, Ppm1d plays a role in the maintenance of hematopoietic stem cells, promotes T- and B-cell development and restricts neutrophil proliferation (Shi et al., 2020). Ppm1d hyperactivity also seems to restrict the infiltration of tumors by antitumor neutrophils, suggesting that Ppm1d inhibition may enhance the effects of cancer immunotherapy (Uyanik et al., 2021). Owing to the function of PPM1D as an oncogene and the relatively mild phenotype of PPM1D depletion in adult mice (Uyanik et al., 2021), PPM1D inhibitors are being evaluated as anti-cancer drugs. The improvement of the pharmacokinetic properties of PPM1D inhibitors will likely lead to clinical trials on PPM1D inhibition in cancer (Deng et al., 2020). Unraveling the spectrum of PPM1D substrates can help to identify common features that underlie the recognition of these proteins by PPM1D and can also provide an unbiased view of its roles in the regulation of the DDR. Furthermore, mapping of the phosphoproteome induced by etoposide and PPM1D inhibition may provide insights into the mechanisms driving the positive selection of cancer cells with PPM1D amplification.

We employed the chemical inhibition of PPM1D with quantitative phosphoproteomics to identify the substrate spectrum of PPM1D after DSB induction by etoposide in human osteosarcoma (U2OS) and colorectal cancer (HCT116) cells. U2OS and HCT116 cells, in addition to wild-type PPM1D, express a truncated version with increased stability leading to increased activity of PPM1D in these cell lines (Kleiblova et al., 2013). We find that PPM1D selectively dephosphorylates 15% of etoposide-induced phosphorylation sites and only partially counteracts ATM/ATR/DNA-PK signaling. PPM1D-targeted sites are found in a specific sequence motif characterized by poor evolutionary conservation, glutamic acid residues, and an intrinsic disorder that is higher than normally observed for phosphorylation site motifs. Furthermore, PPM1D substrates contain DNA- and RNA-binding domains and play a role in DNA repair, transcription regulation, and chromatin organization. We provide evidence that a poorly characterized protein Kanadaptin with an FHA domain and a predicted dsRNA-binding domain is a substrate of ATM and PPM1D and hence may play a role in the DDR.

## Results

### Phosphorylation signaling upon acute double-strand break induction by etoposide is counteracted by PPM1D

**Figure 1:**
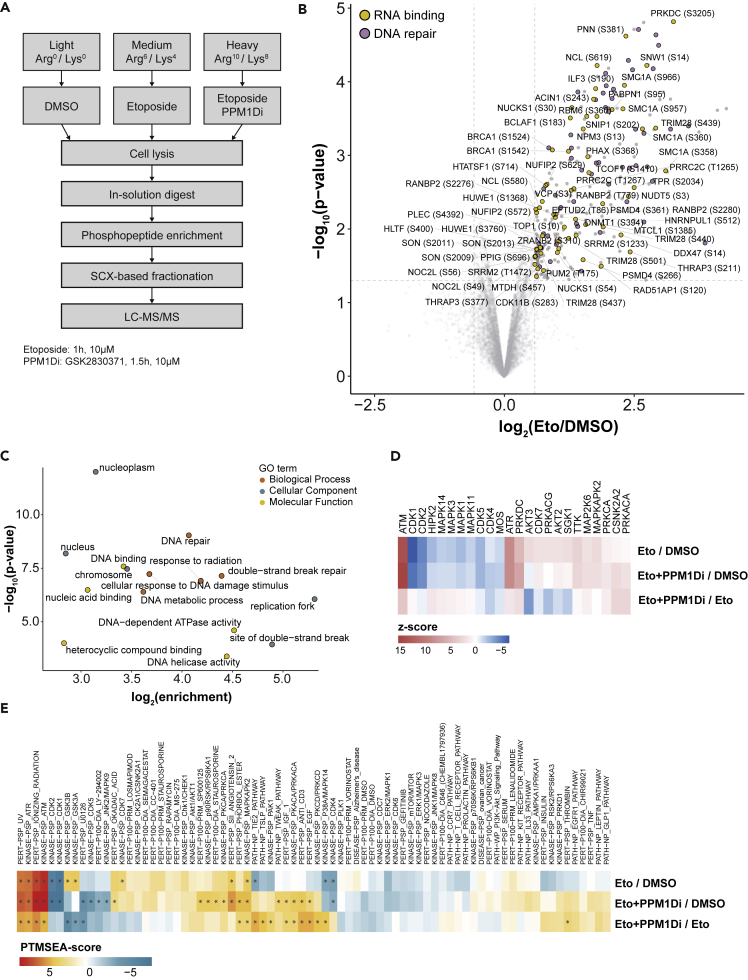
Phosphoproteomic analysis of the etoposide-induced and PPM1D-dependent DNA damage response (A) Schematic representation of the strategy used for phosphoproteomic analysis. Light-, medium- or heavy-labeled U2OS cells were treated either with DMSO, with 10μM etoposide for 1h or with 10μM PPM1D inhibitor for 1.5h followed by etoposide treatment. Cells were lysed and digested using trypsin followed by TiO_2_-based phosphopeptide enrichment and LC-MS/MS analysis. The experiment was performed in triplicates. (B) Volcano plot showing upregulated phosphorylation sites after etoposide treatment (fold change (FC) > 1.5, moderated t-test: p value < 0.05). Phosphorylation sites on proteins mapping to the GO terms DNA repair and RNA binding are highlighted and RNA binding proteins are labeled. Phosphorylation sites with an FC below −2.5 are not shown. (C) GO term analysis of upregulated phosphorylation sites after etoposide treatment using ViseaGO R package (Fisher exact test: p value < 0.05). (D) Kinase-substrate enrichment analysis of etoposide-induced and PPM1D-dependent phosphorylation sites. Relative *Z* score indicates changes in kinase activities after indicated treatments (One-tailed probability test: p value < 0.05). (E) PTM set enrichment analysis showing phosphorylation site-specific pathways, perturbations, and kinase activities (Kolmogorov-Smirnov test: ∗ Benjamini-Hochberg adj. p value < 0.05).

To identify the substrates of PPM1D, we metabolically labeled human osteosarcoma (U2OS) cells with amino acids in cell culture (SILAC). Medium-labeled cells were treated with 10 μM etoposide for 1 h for the acute induction of DSBs. To determine PPM1D substrates upon etoposide treatment, heavy-labeled cells were pretreated with the specific PPM1D inhibitor GSK2830371 (later referred to as PPM1Di) for 30 min and then etoposide was added in the presence of the PPM1Di for 1 h. Phosphorylated peptides were enriched using a titanium dioxide affinity matrix and the enriched peptide fractions were analyzed on a quadrupole-orbitrap hybrid mass spectrometer (Figure 1A and Table S1).

We performed three independent biological replicates to gain a robust quantification of phosphorylation sites and to allow statistical evaluation of the data. Significantly regulated phosphorylation sites were identified using a moderated t-test (limma). Based on these analyses, we identified 270 phosphorylation sites that showed increased abundance in cells treated with etoposide compared to untreated cells (Table S1). The topoisomerase II (TOP2) poison etoposide is widely used in the clinic as an anti-cancer drug (Nitiss, 2009). Etoposide induces DSBs in a replication- and transcription-dependent manner (Tammaro et al., 2013; Gothe et al., 2019). Proteins with etoposide-induced phosphorylation sites included core DSB response factors such as ATM, 53BP1, BRCA1, and CHEK2 as well as proteins with RNA- and DNA-binding domains (Figure 1B and Table S2). These proteins contain etoposide-induced phosphorylation sites and are thus likely involved in the DSB-induced regulation of cellular processes beyond DNA repair. Using gene ontology (GO) enrichment of proteins that show an increase in phosphorylation, we investigated which biological processes are regulated upon etoposide treatment (Figure 1C). The analysis revealed a significant enrichment of the GO biological process terms- DNA repair, cellular response to DNA damage stimulus, double-strand break repair, response to radiation, and DNA metabolic process. We also observed significant enrichment of the GO molecular function terms DNA binding, nucleic acid binding, and DNA−dependent ATPase activity and the GO cellular compartment terms nucleus, nucleoplasm, chromosome, site of double-strand break, and replication fork (Figure 1C).

The phosphatase PPM1D counteracts DNA damage-induced phosphorylation signaling (Lu et al., 2005). To investigate in an unbiased manner how PPM1D inhibition affects protein kinase-dependent signaling, we carried out Kinase-substrate enrichment analysis (KSEA) (Figure 1D) (Casado et al., 2013). As expected, etoposide treatment led to the phosphorylation of substrates dependent on the ATM, ATR, and DNA-PK activity (Figure 1D) (Beli et al., 2012). The signature of cyclin-dependent kinases (CDKs) was reduced, pointing to the induction of DNA damage-induced cell-cycle arrest by etoposide (Donzelli and Draetta, 2003). Inhibition of PPM1D further increased the PIKK signature, which demonstrates that PPM1D antagonizes protein phosphorylation by ATM/ATR/DNA-PK on a significant number of substrates (Figure 1D). To gain further insights into the remodeling of etoposide-induced signaling after PPM1D inhibition, we performed PTM Signature Enrichment Analysis (PTMSEA) (Figure 1E) (Krug et al., 2019). Apart from an increase in PIKK signaling upon inhibition of PPM1D, we found that the signature of the GSK3 signaling is reduced after PPM1D inhibition (Figure 1E). This was also confirmed by examination of sites on individual GSK3B substrates, such as the phosphorylation of MYC at T58 and JUNB at S251, which showed strongly reduced phosphorylation after PPM1D inhibition (Figure S1A). These phosphorylation sites share a functional similarity, leading to FBXW7-mediated proteasomal degradation of the substrate (Yada et al., 2004; Pérez-Benavente et al., 2013). In summary, inhibition of PPM1D leads to a perturbation of DSB-induced phosphorylation pathways that are characterized by a more pronounced PIKK signaling signature.

### PPM1D substrates are nucleic acid-binding proteins that are primarily phosphorylated by ATM upon etoposide treatment

**Figure 2:**
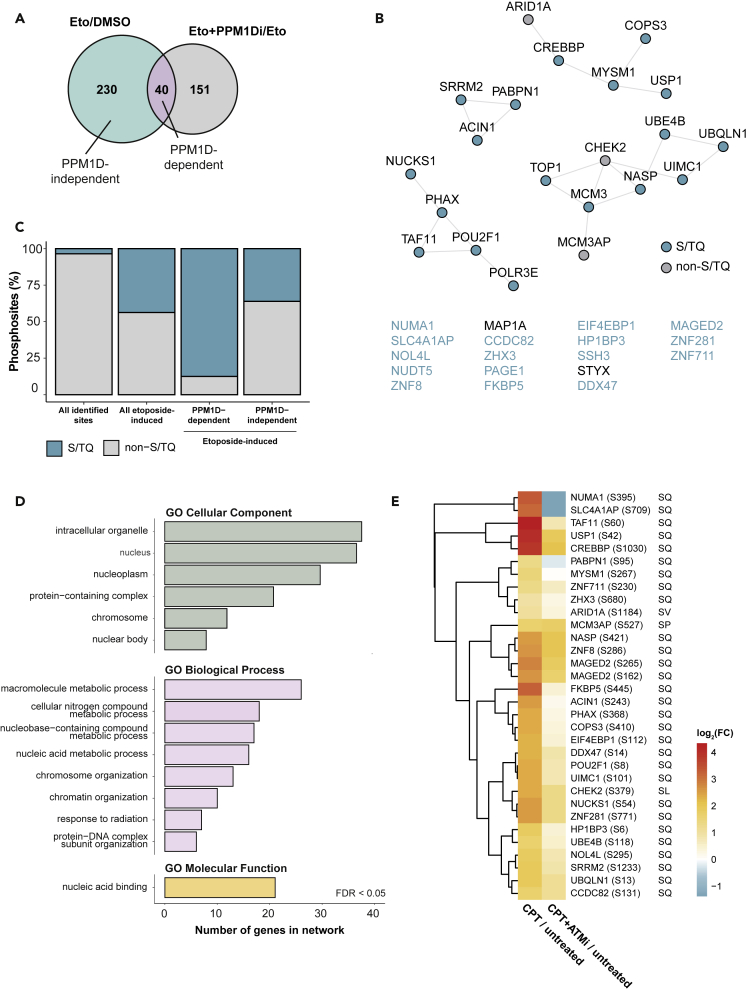
PPM1D-dependent phosphoproteome after DSB induction (A) Etoposide-induced phosphorylation sites were overlapped with sites that show further increase after PPM1Di to determine PPM1D-dependent phosphorylation sites. (B) STRING interaction network (confidence score >0.4) of proteins containing etoposide-induced PPM1D-dependent phosphorylation sites. Sites with an S/TQ motif are annotated in blue. Proteins with no predicted interactions are listed at the bottom. (C) Fractions (%) of S/TQ motif abundance in all identified sites compared to the etoposide-induced, PPM1D-dependent, and PPM1D-independent subset. (D) GO term analysis of etoposide-induced PPM1D-dependent sites curated from the STRING database (FDR (Benjamini-Hochberg method) < 0.05). (E) Heatmap displaying log_2_-transformed FCs of 32 identified PPM1D substrates in response to CPT treatment and combination of CPT and ATMi. FCs were obtained from Balmus et al. (2019). Phosphorylated amino acids and the +1 residue are annotated for each site.

Of the 270 sites on 202 proteins that showed an increased abundance after etoposide treatment, 40 sites (14.8%) on 39 proteins showed a further increase upon PPM1D inhibition and these proteins were considered as etoposide-induced PPM1D substrates (Figures 2A, S2A, and S2B, and Table S3). 118 out of 270 sites occur on serine (114) or threonine (4) followed by a glutamine (S/TQ), a sequence motif recognized by ATM, ATR, and DNA-PK. We found that the inhibition of PPM1D led to increased phosphorylation of 35 of these SQ sites (Figures 2B, 2C, and S2C), suggesting that PPM1D regulates approximately one-third of ATM/ATR/DNA-PK substrates.

We next focused on the nature of the 39 proteins that show an increase in phosphorylation after PPM1D inhibition and are hence putative PPM1D substrates. GO terms analysis revealed that PPM1D substrates are involved in chromatin organization and harbor a nucleic acid-binding molecular function (Figure 2D). Specifically, 21 out of 40 PPM1D substrates are characterized by the presence of an RNA- or DNA-binding region and TOP1 possesses both an RNA- and a DNA-binding region.

To gain insights into whether PPM1D substrates are phosphorylated by ATM, ATR, or DNA-PK, we analyzed a previously published dataset from our group that determined ATM-dependent signaling in HEK293T cells after a short-term treatment with the DSB-inducing TOP1 poison camptothecin (Balmus et al., 2019). Similar to etoposide, camptothecin also induced phosphorylation of all 32 PPM1D substrates co-identified in U2OS and HEK293T cells, pointing to a similarity of the etoposide- and camptothecin-induced DDR. Inhibition of ATM led to a decrease in the phosphorylation of 20 out of 32 putative PPM1D substrates (Figure 2E), suggesting that ATM, and not DNA-PK or ATR, is the main kinase phosphorylating PPM1D substrates in response to DSB induction. Upon ATM inhibition, the strongest decrease in phosphorylation was observed for the previously characterized ATM target NUMA1 (S395) (Bensimon et al., 2020) as well as for the poorly characterized protein Kanadaptin (SLC4A1AP; S709). Among the proteins with decreased phosphorylation upon ATM inhibition were also the previously described substrate ACIN1 (S243) (Bensimon et al., 2020) and the auto-phosphorylated residue of CHEK2 (S379) (Lovly et al., 2008), which acts downstream of ATM.

### PPM1D-regulated phosphorylation sites are located in poorly conserved, glutamic acid-rich intrinsically disordered regions

**Figure 3:**
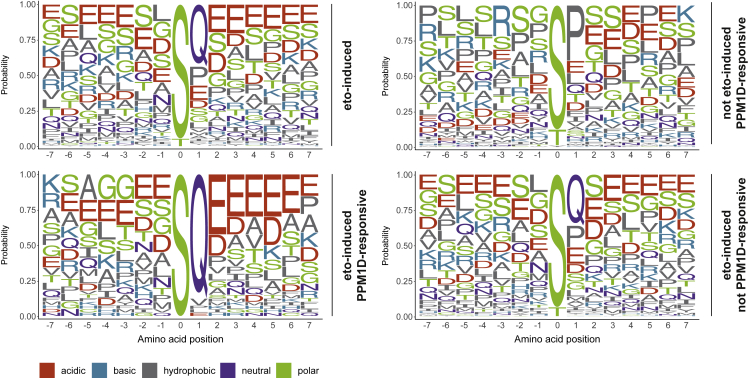
PPM1D substrate motif after DNA damage Sequence motif analysis (+/−7 amino acids) of etoposide-induced and/or PPM1Di-responsive phosphorylation sites. Amino acid probabilities are plotted using the ggseqlogo R package.

**Figure 4:**
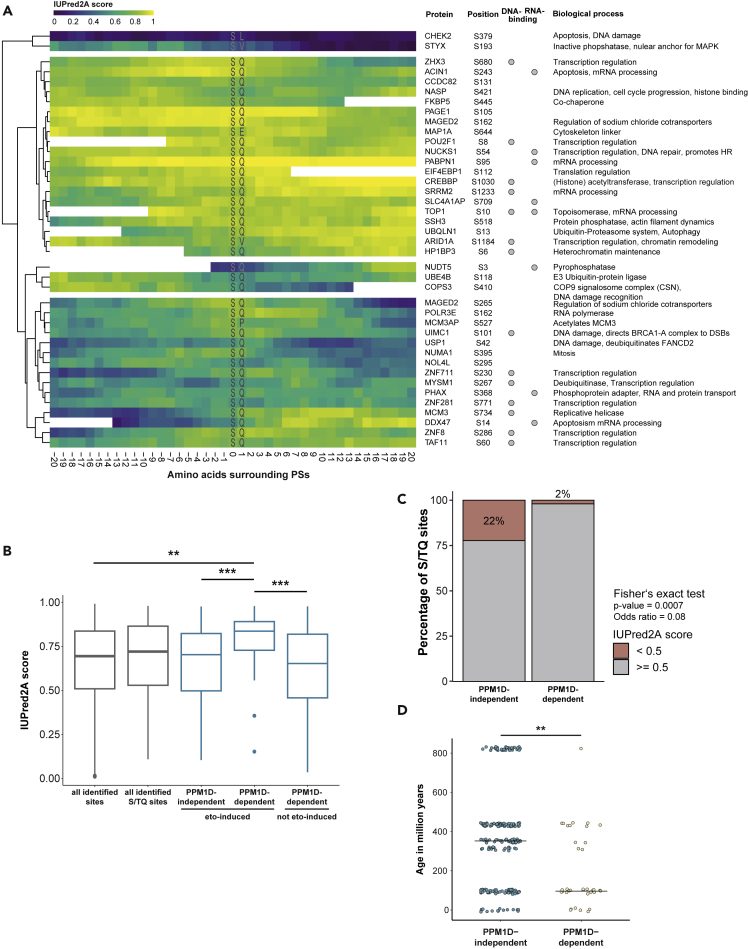
DNA damage-induced PPM1D substrates are located in intrinsically disordered protein regions (A) Intrinsic disorder score (IUPred2A) of surrounding protein regions of etoposide- and PPM1D-dependent phosphorylation sites. IUPred2A score above 0.5 is considered as disorder. Known DNA- and RNA-binding motifs and biological processes of phosphorylated proteins are annotated. (B) Comparison of IUPred2A score of different subsets of etoposide- and PPM1D-targeted sites with S/TQ-motif sites and all identified phosphorylation sites from the phosphoproteome (T-test: ∗∗ p value < 0.001, ∗∗∗ p value < 0.0001). (C) Barplot showing the fraction of S/TQ sites with IUPred2A score <0.5 (not disordered) within PPM1D-dependent sites (upregulated in H/M condition) and PPM1D-independent sites (not upregulated in H/M condition) regardless of their regulation status after etoposide treatment. Fisher's exact test was carried out on the contingency table of S/TQ site counts in each subset. (D) Comparison of estimated phosphorylation site age (+/− 3 amino acids) based on ptmAge prediction of etoposide-induced and PPM1Di-responsive sites with etoposide-induced and PPM1D-independent sites (Cochran-Armitage trend test: ∗∗ p value < 0.001). Datapoints are jittered.

To identify determinants of PPM1D substrate specificity, we performed sequence motif analysis of DSB-induced phosphorylation sites as well as of the sites targeted or not targeted by PPM1D. Treatment of U2OS cells with etoposide led to an increased frequency of sites conforming to the S/TQ motif, which is the consensus motif for the PIKKs ATM, ATR, and DNA-PK (Figure 3A). The distribution of S/TQ-containing phospho-sites showed a skew toward upregulation upon the inhibition of PPM1D, supporting that PPM1D counteracts the phosphorylation of these sites (Figure S2D). The sequence motif analysis of PPM1D-targeted sites revealed enrichment of glutamic acid residues surrounding the phosphorylated serine residue (Figure 3A). Interestingly, sites dependent on PPM1D but not induced by etoposide did not show enrichment of the same motif, suggesting that they are not directly dephosphorylated by PPM1D. Glutamic acid residues are thought to promote intrinsic disorder (Campen et al., 2008) and are often enriched within intrinsically disordered regions (IDRs) (Bürgi et al., 2016). To investigate whether etoposide-regulated PPM1D-targeted sites are enriched in IDRs, we annotated the proteins with the site-specific disorder score using the IUPred2A algorithm (Mészáros et al., 2018). This revealed that PPM1D-targeted sites are predominantly located in IDRs (IUPred2A score >0.5) (Figure 4A). Importantly, the predicted intrinsic disorder was often not confined only to the phosphorylated residue and the surrounding few residues, but extended well beyond in both upstream and downstream directions, implying that these phospho-motifs could, indeed, be localized in larger IDRs. To further explore this possibility, we mapped the phosphorylation sites of 6 selected proteins with a high IUPred2A score (PABPN1, EIF4EBP1, CREBBP, TOP1, UBQLN, and NUCKS1) onto the predicted Alpha-Fold structures (Jumper et al., 2021). This analysis revealed that phosphorylated serines of all tested proteins localize to regions high in disorder (Figure S3A).

To statistically test the enrichment of intrinsic disorder in PPM1D-targeted motifs, we compared IUPred2A scores of PPM1D-targeted motifs with the motifs found in all quantified sites or in all quantified S/TQ sites. This comparison revealed that PPM1D-targeted motifs display higher intrinsic disorder than the ones found in S/TQ motifs that are not targeted by PPM1D (Figure 4B). Although 22% of PPM1D-independent S/TQ sites have an IUPRED2 score below 0.5 and are thus likely found within structured domains, only 2% of PPM1D-dependent S/TQ sites fall within the same category (Figure 4C).

IDRs have high mutation rates and thus play a central role in the evolution of regulatory signaling cascades (Nilsson et al., 2011). We, therefore, explored whether etoposide-induced PPM1D-targeted motifs differ from PPM1D-independent, etoposide-induced motifs in their conservation by comparing their evolutionary age. We found that, among etoposide-induced sites, PPM1D-targeted phosphorylation sites are significantly less conserved in the evolution compared to the phosphorylation sites not targeted by PPM1D (Figure 4D). The same is true for all S/TQ phosphorylation sites, 31% of which are younger than 200 million years among sites not targeted by PPM1D and 58% among PPM1D-targeted sites (Figure S3B).

### Comparison of the substrate landscape of PPM1D in HCT116 and U2OS cells

**Figure 5:**
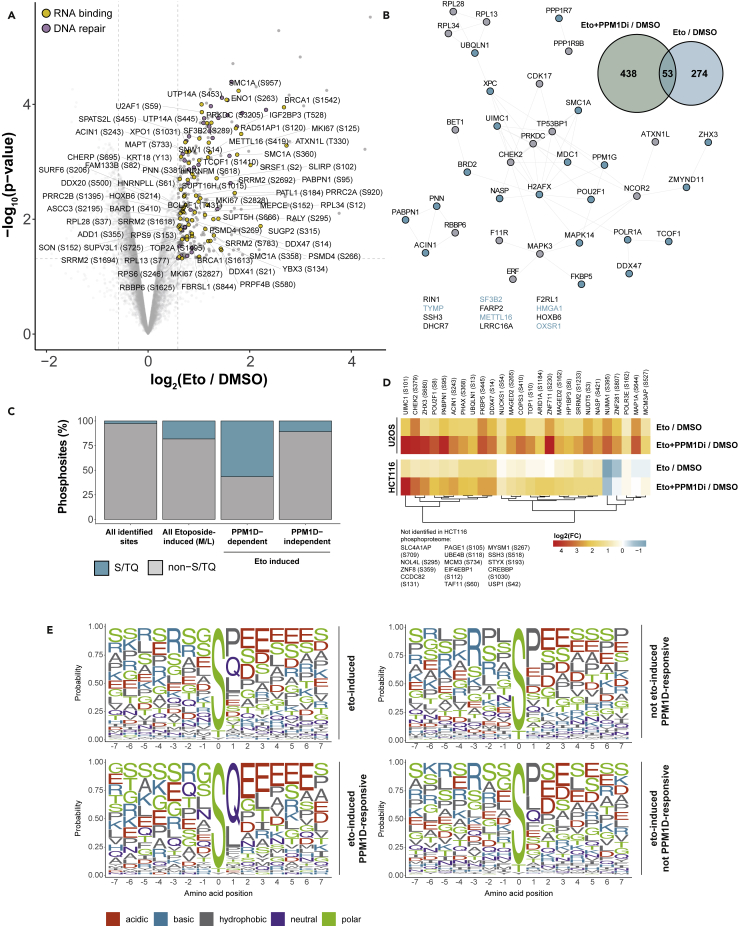
Phosphoproteomic analysis of PPM1D substrates after etoposide treatment in HCT116 cells (A) Volcano plot of phosphorylation sites after etoposide treatment (FC > 1.5, moderated t-test: p value < 0.05). Phosphorylation sites on proteins mapping to the GO terms DNA Repair and RNA binding are highlighted and RNA binding proteins are labeled. (B) Etoposide-induced site was overlapped with upregulated sites after combined etoposide and PPM1Di treatment compared to etoposide treatment. Network showing proteins containing the 53 overlapping phosphorylation sites (STRING conf. score >0.4). Sites containing an SQ motif are colored in blue and proteins without any known interaction partner in the network are shown below. (C) Fractions (%) of S/TQ motif abundance in all identified sites compared to the etoposide-induced, PPM1D-dependent, and PPM1D-independent subset. (D) Log_2_ fold changes in HCT116 screen of etoposide- and PPM1Di-induced phosphorylation sites from the U2OS screen. Sites that are not identified in the HCT116 screen are annotated aside. (E) Sequence motif analysis of sites belonging to the different subsets from the HCT116 screen. Amino acid probabilities are plotted using the ggseqlogo R package.

To investigate the generalizability of the identified PPM1D substrates beyond U2OS cells, we repeated the same phosphoproteomics screen in human colorectal cancer HCT116 cells (Figure 5A and Table S4). We identified 53 putative PPM1D substrates induced by etoposide using the same fold change and significance cutoffs (Figure 5B), among which were core DNA damage response proteins H2AX (S139/140), MDC1 (S1086), 53BP1 (S834, S1320), CHEK2 (S159), DNA-PK/PRKDC (S3205) and RAP80/UIMC1 (S101). Etoposide also caused an increase in S/TQ phosphorylation in HCT116, and this was even more pronounced upon PPM1D inhibition (Figure 5C). A comparison of treatments revealed significant overlaps between U2OS and HCT116 cells: 26 out of 40 PPM1D-targeted sites were also quantified in HCT116 cells and these sites followed the same trend, displaying increased phosphorylation after etoposide and a further increase after PPM1D inhibition (Figure 5D). Only four proteins (NUMA1, ZNF281, MAP1A, and MCM3AP) identified in U2OS cells were not phosphorylated upon etoposide treatment in HCT116 cells. We identified 21 additional etoposide-induced PPM1D substrates targeted on the S/TQ motif in HCT116 (Figures S4A and S4B) with a mean IUPRED2 score of 0.81. Etoposide-induced PPM1D-targeted sites also showed an enrichment of glutamic acid residues surrounding the phosphorylation site in HCT116 cells (Figure 5E).

Kinase-substrate enrichment analysis in HCT116 cells confirmed the effect of etoposide and PPM1D inhibition on ATM and CDK signaling (Figure S4C). Additionally, multiple cytoplasmic kinase pathways were upregulated by the inhibition of PPM1D independently of DNA damage. This was predominant for MAPK pathways, with MAPKAPK2 (MK2) being induced by PPM1D inhibition in unchallenged cells. p38/MAPK14, a kinase upstream of MK2, has been extensively studied in relation to PPM1D (Bulavin et al., 2004; Demidov et al., 2007; Liu et al., 2013). It was shown that PPM1D represses p38 activity, possibly by direct dephosphorylation of p38 at T180/Y182 (Takekawa et al., 2000). We, therefore, investigated MK2/3/5 substrates previously identified by our group (Borisova et al., 2018) as well as p38 and MK2 substrates retrieved from PhosphoSitePlus and found evidence that p38-MK2 signaling is increased after the inhibition of PPM1D in unchallenged cells (Figure S4D). MK2 is known to phosphorylate substrates at the RxxS/T motif (Borisova et al., 2018). Accordingly, we found enrichment of arginine residues at the −3 position from the phospho-site in both U2OS (Figure 3A) and HCT116 (Figure 5E) among PPM1D-dependent sites in unchallenged cells.

### Phosphorylation of the poorly characterized protein Kanadaptin by ATM is counteracted by PPM1D

**Figure 6:**
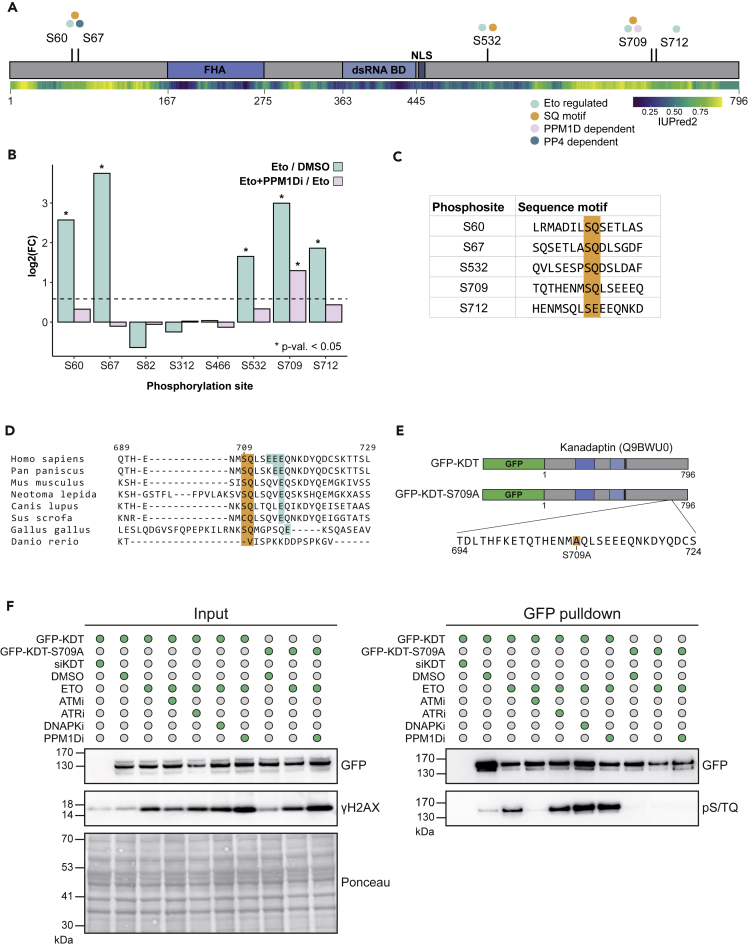
Kanadaptin is phosphorylated by ATM and dephosphorylated by PPM1D in response to DSBs (A) Scheme of human Kanadaptin (Q9BWU0) containing an FHA domain, SMART-predicted double-stranded RNA binding domain, and nuclear localization signal (NLS). Regulated phosphorylation sites after etoposide and PPM1Di treatment are annotated with their dependencies and SQ sites are indicated. Regulation by PP4 is predicted based on (Ueki et al., 2019) IUPred2A score is mapped to the protein and represents intrinsic disorder. (B) log_2_-transformed FCs of all Kanadaptin phosphorylation sites identified by phosphoproteomics (moderated t-test: ∗ p value < 0.05). (C) Amino acid environment of significantly upregulated Kanadaptin phosphorylation sites after DNA damage. (D) Multiple sequence alignment (ClustalW) of pS709/S712 motif. Phosphorylated serine +1 amino acid and downstream glutamic acid residues are highlighted. (E) Scheme of GFP tagged wt-Kanadaptin and phospho-dead (S709A) constructs. (F) GFP-Kanadaptin or GFP-Kanadaptin-S709A were transiently expressed in U2OS cells treated with 10μM etoposide for 1h, or additionally treated with ATMi (10μM), ATRi (2μM), PPM1Di (10μM), or DNAPKi (10μM) for 1.5h, or co-transfected with siRNA against Kanadaptin. GFP-Kanadaptin was pulled down followed by washes in 8M urea.

Among the proteins with phosphorylation sites induced after treatment with etoposide, we identified a functionally uncharacterized protein Kanadaptin. It was initially misannotated as an interactor of kidney anion transporter 1 (SLC4A1) and given the gene name *SLC4A1AP.* Later experiments showed that Kanadaptin does not interact with SLC4A1 nor is involved in its targeting of the plasma membrane (Kittanakom et al., 2004). Interestingly, Kanadaptin contains an FHA domain as well as a putative dsRNA-binding domain followed by a nuclear localization signal (NLS) (Figure 6A). In line with this, its interphase localization is predominantly nuclear (Hübner et al., 2002).

Etoposide treatment induced phosphorylation of Kanadaptin on five serine residues, four of which are SQ sites (Figures 6A and 6C), indicative of phosphorylation by ATM, ATR, or DNA-PK. One of the etoposide-induced SQ sites, S709, showed an increase after PPM1D inhibition (Figure 6B), suggesting that PPM1D is responsible for its dephosphorylation. Similar to other PPM1D substrates, three glutamic acid residues are found downstream of S709 and this residue is located in a region predicted to be highly intrinsically disordered (Figures 6A and S5A). Furthermore, the SQ motif of S709 is conserved from birds to humans, but the appearance of multiple glutamic acid residues is relatively recent, with apes and humans each evolving an additional glutamic acid residue downstream of the SQ motif (Figure 6D).

To further validate the dependence of the S709 phosphorylation status on PIKKs and PPM1D, we transiently expressed GFP-tagged WT Kanadaptin or the phospho-dead S709 mutant (GFP-KDT-S709A) in U2OS cells that were either mock-treated, treated with etoposide alone or in combination with ATM, ATR, DNA-PK or PPM1D inhibitor (Figure 6E). We then performed a pulldown using GFP-Trap agarose followed by stringent washes to ensure Kanadaptin is the predominant immunoprecipitated protein. We analyzed the eluate by Western blot and confirmed that the phosphorylation of Kanadaptin at the SQ motif increases after etoposide treatment using the pS/TQ motif antibody (Figure 6F). Moreover, the pS/TQ signal was abolished after ATM inhibition and not after the inhibition of ATR or DNA-PK. Interestingly, inhibition of DNA-PK increased phosphorylation of Kanadaptin, likely because DNA-PK can restrict ATM activity (Finzel et al., 2016). These results demonstrate that ATM is the primary kinase phosphorylating Kanadaptin at the SQ residues upon acute etoposide treatment (Figure 6F). PPM1D inhibition increased the etoposide-induced pSQ signal, confirming that PPM1D dephosphorylates at least one of the SQ residues on Kanadaptin. Mutation of S709 to alanine almost completely abolished the SQ phosphorylation, suggesting that S709 is the stoichiometrically predominant Kanadaptin residue phosphorylated upon etoposide treatment or acts as a priming event enabling the phosphorylation of other SQ motifs (Figure 6F).

**Figure 7:**
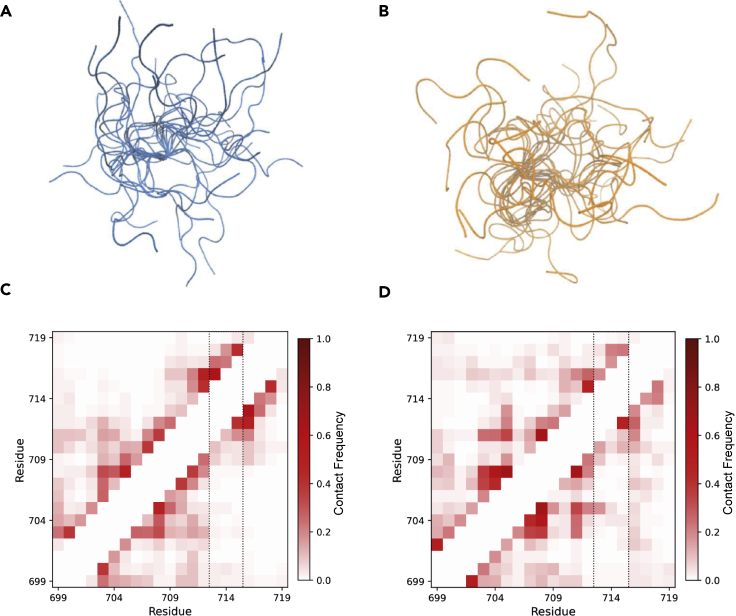
Conformational flexibility of (un)phosphorylated Kanadaptin SQ motif (A and B) Visualization of conformational ensembles of unphosphorylated and phosphorylated SQ motifs. Simulation structures were aligned on central amino acids M708 and S709. (C and D) Contacts in the simulation of unphosphorylated and phosphorylated SQ motifs. Contact maps show the frequency of contacts between pairs of residues in the MD simulation trajectories. Triple glutamate motif is highlighted with dashed lines.

In order to characterize the structure-function and disorder-function relationships of the disordered SQ motif of Kanadaptin (around S709), we ran μs long atomistic molecular dynamics (MD) simulations, which also enabled us to explore the possible effects of phosphorylation on this sequence motif. The SQ motif is highly enriched in both negatively and positively charged side-chains and based on these sequence characteristics is considered a polyampholyte and can in principle adopt both extended structures and hairpin-like conformations (Das and Pappu, 2013). Such structural propensities can be resolved accurately in atomistic molecular dynamics simulations (Robustelli et al., 2018; Pietrek et al., 2020; Stelzl et al., 2022). Overall, the 21 amino acid motif stays disordered in the MD simulations, whether its central serine is unphosphorylated (Figure 7A) or phosphorylated (Figure 7B). The motif adopts mostly extended or hairpin-like conformations in the MD simulations. Radius of gyration (R_G_), a measure of extension, does not drop during the simulations as no collapsed conformations are populated (Figure S6A). R_G_ values are close to the value of 1.19 nm predicted for a prototypical unfolded protein segment of this length (Kohn et al., 2004). Some tendency to form helical-like structures is apparent around the central serine residue in the simulation with the phosphorylated and unphosphorylated SQ motif and the three glutamate residues in the simulations of the unphosphorylated SQ motif (Figures S6B and S6C). However, most residues interact with adjacent residues or with the solvent. The phosphorylation of S709 increases the solvent-accessible surface area of S709 (Figure S6D) and reduces the contact frequency between S709 and the close by residues (H 705 and E 706) and between E713 and the adjacent residues (Q 716 and N 717) (Figure S6E). There are few long-range contacts (Figures 7C and 7D). For the phosphorylated protein, there is a weakly populated contact between pS709 and K700 and a transient contact between T705 and E715, but typically the glutamate side chains interact with the solvent (Figures S6F and S6G). As the three glutamic acid residues (E 713, 714, and 715) are mostly solvent-exposed throughout the simulations, they might have a role in molecular recognition and protein-protein interactions of the PPM1D-targeted SQ motif in the DDR.

## Discussion

We employed chemical inhibition with quantitative phoshoproteomics to interrogate the substrate spectrum of the phosphatase PPM1D in response to acute DSB formation induced by the TOP2 poison etoposide. We identified 35 putative substrates of PPM1D in U2OS cells and an additional 21 in HCT116 cells that are phosphorylated on serine residues followed by a glutamine (consensus SQ motif targeted by ATM/ATR/DNA-PK). Many PPM1D substrates associate with chromatin and more than half contain predicted RNA- and/or DNA-binding regions. We demonstrated that upon acute induction of DSBs, PPM1D counteracts the phosphorylation of one-third of ATM/ATR/DNA-PK substrates. The majority of these sites are phosphorylated by ATM, in line with the predominant role of this kinase after DSB induction. This suggests that PPM1D inhibits ATM signaling at two levels: firstly, by dephosphorylating ATM itself (Shreeram et al., 2006), and secondly, by dephosphorylating a fraction of ATM substrates.

Two previous studies employed quantitative proteomics to analyze changes in the phosphoproteome upon PPM1D inhibition. Kahn et al. treated the wild-type and PPM1D-mutant AML cell line Molm13 with the antimetabolite cytarabine and assessed the effects of the specific PPM1D inhibitor GSK2830371 in these cells (Kahn et al., 2018). Upon inhibition of PPM1D, they observed increased phosphorylation of DNA repair factors including p53, CHEK1, and CHEK2 and concluded that PPM1D is a major regulator of the DDR.

Wei et al. compared the phosphoproteomes in testes from wild-type and Ppm1d −/− mice and implicated Ppm1d in the regulation of adherens/tight junctions, apoptosis, inflammatory response, spermatogenesis, sperm motility, and cytoskeletal assembly and depolymerization in this tissue (Wei et al., 2019). Additionally, they showed increased levels of pro-inflammatory cytokines in Ppm1d knockout testes, which was proposed to impair the blood-testis barrier and lead to decreased fertility.

We found that PPM1D substrates are phosphorylated on an SQ motif rich in glutamic acid residues and present in IDRs that show an unusually high intrinsic disorder. Glutamic acid residues are thought to promote intrinsic disorder (Campen et al., 2008) and are often enriched within IDRs (Bürgi et al., 2016). Previous studies have shown that phosphorylation sites are frequently located in IDRs (Iakoucheva et al., 2004; Collins et al., 2008; Bludau et al., 2022). Besides being rich in disorder-promoting residues, intrinsically disordered proteins (IDPs) often contain DNA- or RNA-binding domains (Zhao et al., 2021) and are involved in the regulation of transcription and translation (Liu et al., 2006), as well as protein phosphorylation (Bah and Forman-Kay, 2016). Furthermore, IDPs are known to undergo phase separation mediated by IDRs and RNA-binding domains (Ilık et al., 2020; Schuster et al., 2020). Also, phosphorylation can modulate the properties of protein condensates, including the recruitment or exclusion of proteins from condensates (Monahan et al., 2017). We found that PPM1D substrates associate with chromatin and often contain DNA- or RNA-binding domains. Indeed, there is evidence for phase separation of multiple PPM1D substrates that we identified: SRRM2 is a *bona fide* marker of nuclear speckles (NSs), a non-membrane-bound phase-separated nuclear structure involved in transcriptional processes (Ilık et al., 2020). ACIN1, CREBBP and PABPN1 also localize to NSs (Calado and Carmo-Fonseca, 2000; Von Mikecz et al., 2000; Wang et al., 2014). UBQLN1 is ubiquitin-binding shuttle protein that promotes the degradation of proteins through the ubiquitin-proteasome system and autophagy. UBQLN proteins are mutated in neurodegenerative diseases and cancer and form membraneless organelles in cells (Zheng et al., 2020). It is possible that the phosphorylation of UBQLN1 in its N-terminal IDR regulates its liquid-liquid phase separation (LLPS) behavior and plays a role in targeted protein degradation in response to genotoxic stress. Nucleoli are nuclear compartments showing phase-separation behavior (Frottin et al., 2019). PPM1D was shown to localize to nucleoli and implicated in the nucleolar formation by regulating the phosphorylation of nucleophosmin (Kozakai et al., 2016). Therefore, PPM1D does not only dephosphorylate substrates that can reside within phase-separated compartments, but can also localize to such compartments.

We have demonstrated that a poorly characterized protein Kanadaptin is phosphorylated at multiple SQ motifs upon etoposide-induced DSB by ATM. Notably, Kanadaptin contains an FHA domain that is found in DDR proteins MDC1, NBS1, RNF8, and CHEK2 and a putative dsRNA-binding domain similar to the one in the RNA helicase DHX9. The crystal structure of the FHA domain of Kanadaptin revealed its similarity with other FHA domains and ability to bind phospho-threonine residues (Xu et al., 2014). Notably, Kanadaptin was identified as one of the top interactors of MDC1 in a proximity proteomics screen, further supporting its role in the DDR (Gupta et al., 2018). Our MD simulations of Kanadaptin suggest that the phosphorylation site and the triple glutamate motif engage in few interactions on their own, interact mostly with solvent, and thus may be poised to interact with positively charged groups in interacting proteins. Recent studies have shown that highly charged sequences can phase-separate (Gruijs da Silva et al., 2022), in particular when the numbers of positively and negatively charged side chains are balanced (Bremer et al., 2022). We propose that the phosphorylation of proteins at the S/TQ motif, in particular when surrounded by disorder-promoting residues such as glutamic acid, might be involved in the formation of biomolecular condensates by LLPS.

### Limitations of the study

PPM1D activity is frequently altered in cancer and different mutations have been reported that primarily result in increased phosphatase activity. This study focuses on the identification of PPM1D substrates in cancer cells expressing hyperactive PPM1D and thus we do not exclude the possibility that the substrate spectrum of PPM1D in these cells is altered compared to cells expressing the wild-type protein. PPM1D is known to be activated in response to different types of DNA damage and in this study, we exclusively focused on the substrate spectrum of PPM1D upon acute induction of DSBs.

## STAR★Methods

### Key resources table

**Table:** 

REAGENT or RESOURCE	SOURCE	IDENTIFIER
**Antibodies**		

γH2AX	Bethyl Laboratories Inc.	A300-081A-M; RRID:AB_2779000
GFP	Chromotek	PABG1; RRID:AB_2749857
Phospho-ATM/ATR substrate Motif (pSQ)	Cell Signaling	9607; RRID:AB_10889739
Polyclonal Goat Anti-Mouse Immunoglobulins/HRP	Agilent Technologies	P044701-2; RRID:AB_2617137
Polyclonal Goat Anti-Rabbit Immunoglobulins/HRP	Agilent Technologies	P044801-2; RRID:AB_2617138

**Chemicals, peptides, and recombinant proteins**		

PPM1Di (GSK2830371)	Selleckchem	S7573
ATMi (KU-55933)	Selleckchem	S1092
ATRi (VE-821)	Selleckchem	S8007
DNAPKi (NU7441)	Selleckchem	S2638
Etoposide	Sigma-Aldrich	E1383
Arg-0	Sigma-Aldrich	A6969
Lys-0	Sigma-Aldrich	L8662
Arg-6 isotope	Eurisotop	CLM-2265-H-1
Lys-4 isotope	Eurisotop	DLM-2640-1
Arg-10 isotope	Eurisotop	CNLM-539-H-1
Lys-8 isotope	Eurisotop	CNLM-291-H-1
Lys-C	FUJIFILM Wako Pure Chemical Corporation	4987481427648
Protease inhibitor cocktail	Sigma-Aldrich	P8340-5ML
Trypsin	Serva	37286.01
Titansphere TiO Bulk Material (10 μm)	GL Sciences	5020-75010
Q5 Polymerase	IMB Core Facilities	N/A
T4 Polynucleotide Kinase	New England BioLabs	M0201S
T4 DNA ligase	New England BioLabs	M0202S

**Critical commercial assays**		

QuickStart Bradford Protein assay	BioRad	5000201
SuperSignal™ West Pico PLUS Chemiluminescent Substrate	Thermo Fisher	34579
Immobilon ECL Ultra Western HRP Substrate	Merck	WBULS0100

**Deposited data**		

Etoposide/PPM1Di phosphoproteomics dataset (U2OS and HCT116)	This manuscript	PRIDE: PXD035420

**Experimental models: Cell lines**		

U2OS	ATCC	HTB-96; RRID:CVCL_0042
HCT116	ATCC	CCL-247; RRID:CVCL_0291

**Oligonucleotides**		

ON-TARGETplus Human SLC4A1AP siRNA SMARTpool	Dharmacon	L-021089-00-0010
ON-TARGETplus Non-targeting Control Pool	Dharmacon	D-001810-10-20
Primer: KDT_insert_fwd agcaggcttaATGTTAGCACCACTTCGC	This paper	N/A
Primer: KDT_insert_rev aagctgggttATAGCCATACTTG TCATTAAGATG	This paper	N/A
Primer: KDT_backbone_fwd gtatggctatAACCCAGCTTTCTTGTAC	This paper	N/A
Primer: KDT_backbone_rev gtgctaacatTAAGCCTGCTTTTTTGTAC	This paper	N/A
Primer: KDT_SDM_S709A_fwd TGAAAACATGGCCCAACTTAGCG	This paper	N/A
Primer: KDT_SDM_S709A_rev CGCTAAGTTGGGCCATGTTTTCA	This paper	N/A

**Recombinant DNA**		

MGC Human SLC4A1AP Sequence-Verified cDNA	MHS6278-211690004	Horizon

**Software and algorithms**		

RStudio (v. 4.1)	N/A	RRID:SCR_000432
MaxQuant (v. 1.5.2.8)	Cox and Mann (2008)	RRID:SCR_014485
KSEA algorithm	Casado et al. (2013)	N/A
IUPred2A	Mészáros et al. (2018)	N/A
Clustal Omega	Madeira et al. (2019)	RRID:SCR_001591
Gromacs 2021	Toxvaerd et al. (2012)	N/A
AmberTools	Case et al. (2021)	RRID:SCR_018497

**Other**		

Sep-Pak C18 cartridges	Waters	WAT036945
GFP-trap Agarose	Chromotek	gta-10
NuPAGE® Bis-Tris Precast Gels	Invitrogen	NP0321BOX

### Resource availability

#### Lead contact

Further information and requests for resources and reagents should be directed to and will be fulfilled by the lead contact, Sebastian A. Wagner (swagner@med.uni-frankfurt.de).

#### Materials availability

Plasmids generated in this study are available on request.

### Experimental model and subject details

U2OS (derived from bone tissue from 15-year old female osteosarcoma patient) and HCT116 (derived from the colon of adult male) cells were obtained from ATCC and cultured in D-MEM medium supplemented with 10% fetal bovine serum, L-glutamine, penicillin and streptomycin. Cells were routinely tested for mycoplasma infection with a PCR-based method. For SILAC labeling, cells were cultured in media containing either L-arginine and L-lysine, L-arginine [13C6] and L-lysine [2H4] or L-arginine [13C6-15N4] and L-lysine [13C6-15N2] (Cambridge Isotope Laboratories) as described previously (Ong et al., 2002). All cells were cultured at 37°C in a humidified incubator containing 5% CO2.

### Method details

#### Cell lysis

Cells were lysed in modified RIPA (mRIPA) buffer (50mM Tris-HCL, 150mM NaCl, 1mM EDTA, 1% NP-40, 0.1% Na-deoxycholate, pH 7.5) supplemented with protease and phosphatase inhibitors and incubated for 30 minutes at 4°C on a rotation wheel. Lysates were cleared by centrifugation at 16000 × g and protein concentrations were estimated using QuickStart Bradford Protein assay (BioRad). For PPM1D phosphoproteome, SILAC labeled U2OS or HCT116 cells were pretreated with 10μM PPM1D inhibitor (GSK2830371, Selleckchem) for 30 minutes and subsequently treated with DMSO or 10μM etoposide (Sigma) for 1h before harvesting.

#### Phosphoproteomics sample preparation

Phosphoproteome method was performed as described previously (Borisova et al., 2017). Proteins were precipitated in fourfold excess of ice-cold acetone and subsequently re-dissolved in denaturation buffer (6 M urea, 2 M thiourea in 10 mM HEPES pH 8.0). Cysteines were reduced with 1 mM dithiothreitol (DTT) and alkylated with 5.5 mM chloroacetamide. Proteins were digested with endoproteinase Lys-C (Wako Chemicals) and MS-approved trypsin (Serva). Protease digestion was stopped by addition of TFA to 0.5% and precipitates were removed by centrifugation. Peptides were purified using reversed-phase Sep-Pak C18 cartridges (Waters) and eluted in 50% acetonitrile, 0.1% TFA. Phosphopepetides were enriched by incubation with titanium dioxide spheres (GL Sciences) for 2 × 1h with rotation. They were eluted sequentially with 5% NH4OH and 10% NH4OH 25% ACN, and vacuum concentrated to remove NH4OH. Peptides were separated into ten fractions using micro-column-based SCX and desalted on reversed phase C18 StageTips.

#### MS analysis

Peptide fractions were analyzed on a quadrupole Orbitrap mass spectrometer (Q Exactive Plus, Thermo Scientific) equipped with a UHPLC system (EASY-nLC 1000, Thermo Scientific) as described (Michalski et al., 2011; Kelstrup et al., 2012). Peptide samples were loaded onto C18 reversed phase columns (15 cm length, 75 μm inner diameter, 1.9 μm bead size) and eluted with a linear gradient from 8 to 40% acetonitrile containing 0.1% formic acid in 2 h. The mass spectrometer was operated in data-dependent mode, automatically switching between MS and MS^2^ acquisition. Survey full scan MS spectra (m/z 300–1650) were acquired in the Orbitrap. The 10 most intense ions were sequentially isolated and fragmented by higher-energy C-trap dissociation (HCD) (Olsen et al., 2007). Peptides with unassigned charge states, as well as with charge states less than +2 were excluded from fragmentation. Fragment spectra were acquired in the Orbitrap mass analyzer.

#### Atomistic molecular dynamics simulations

The atomistic simulations of unphosphorylated and phosphorylated Kanadaptin protein fragment ranging from amino acid 699 to 719 were conducted in Gromacs 2021 (Toxvaerd et al., 2012) with the AMBER99SB∗-ILDN-q protein (Hornak et al., 2006; Best and Hummer, 2009; Lindorff-Larsen et al., 2010; Best et al., 2012) and TIP4P-D water model (Piana et al., 2015) as before (Pietrek et al., 2020). Phosphoserine parameters were taken from Homeyer et al. (2006), with a correction to the oxygen parameters by Steinbrecher et al. (Steinbrecher et al., 2012). Coordinates for the phosphoserine were prepared with AmberTools (Case et al., 2021). The unphosphorylated and phosphorylated groups were each solvated in water with 150 mM NaCl to neutralize the system. Each simulation system contained more than 35000 atoms. The Particle Mesh Ewald method was used to treat long-range electrostatics. Van-der-Waals interactions were cut off at 12 Å. 600 ps equilibration was performed before production MD runs of over 130 ns. Production simulations were run in the NPT ensemble with the temperature and pressure maintained at 300K and 1 bar with the Bussi-Donadio-Parrinello velocity-rescaling thermostat (Bussi et al., 2007) and Parrinello-Rahman barostat respectively (Parrinello and Rahman, 1981). Simulations were analyzed with Gromacs and the mdtraj Python library (McGibbon et al., 2015). Contact maps were computed using https://contact-map.readthedocs.io/. Simulation trajectories were visualized by VMD software (Humphrey et al., 1996). To visualize the diversity of the ensembles we loaded 5000 confirmations from 500 ns to 1 μs drawing every 300^th^ structures.

#### Site-directed mutagenesis of KDT

To produce phospho-dead mutant (S709A) of KDT, site-directed mutagenesis was performed on pENTR221-KDT vector. Plasmids were amplified by PCR using Q5 polymerase (IMB Core Facilities) and mutant-specific primers (see key resources table). 5′-phosphorylation was done with T4 Polynucleotide Kinase (NEB) for 30 minutes at 37°C. Subsequent ligation was carried out by T4 DNA ligase (NEB) for 2h at RT. Cloning results were validated by sequencing (Eurofins Genomics). LR reaction was used to generate GFP-tagged KDT, results were confirmed by sequencing.

#### ATM/ATR substrate phosphorylation assay

U2OS cells were co-transfected with GFP-KDT and lysed 48h after transfection as described above. For KDT knockdown, cells were initially transfected with siKDT pool (Dharmacon). Cells were treated with 10μM etoposide for 1h or additionally treated with 10μM of ATMi (KU-55933, Selleckchem), 2μM ATRi (VE-821, Selleckchem), 10μM DNAPKi (NU-7441, Selleckchem) or 10μM PPM1Di for 1.5h. 30μl of GFP-trap Agarose (Chromotek) beads were washed three times with RIPA buffer and incubated with the cell lysates for 1 hour at 4°C on a rotation wheel. Beads were washed three times with 8M Urea, 1% SDS in PBS and one time with 1% SDS in PBS.

#### SDS-PAGE and western blotting

Proteins were resolved on 4-12% gradient SDS-PAGE gels (NuPAGE® Bis-Tris Precast Gels, Life Technologies) and transferred onto nitrocellulose membranes. Membranes were blocked using 10% skimmed milk solution in PBS supplemented with 0.1% Tween-20. The list of antibodies used in this study and conditions can be found in key resources table. Secondary antibodies coupled to horseradish peroxidase (Agilent Technologies) were used for immunodetection. The detection was performed with SuperSignal West Pico Chemiluminescent Substrate (Thermo Scientific) or Immobilon ECL Ultra-Western HRP Substrate (Merck).

### Quantification and statistical analysis

#### Peptide identification

Raw data files were analyzed using MaxQuant (development version 1.5.2.8) (Cox and Mann, 2008). Parent ion and MS^2^ spectra were searched against a database containing 92,607 human protein sequences obtained from the UniProtKB released in 05/2016 using Andromeda search engine (Cox et al., 2011). Spectra were searched with a mass tolerance of 6 ppm in MS mode, 20 ppm in HCD MS^2^ mode, strict trypsin specificity and allowing up to 2 miscleavages. Cysteine carbamidomethylation was searched as a fixed modification, whereas phosphorylation (STY), protein N-terminal acetylation and methionine oxidation were searched as variable modifications. The dataset was filtered based on posterior error probability (PEP) to arrive at a false discovery rate of below 1% estimated using a target-decoy approach (Elias and Gygi, 2007).

#### Computational analysis of MS data

Processed data was analyzed using RStudio software environment (version 4.1). Identified peptides were filtered for potential contaminants, reverse reads and localization probability (>75%). P-values were calculated by a moderated t-test using LIMMA package (Ritchie et al., 2015). For PPM1D-dependent phosphoproteome only regulated phosphorylation sites with a p-value < 0.05 were considered as significant. For network analysis, protein-protein interactions were obtained from STRING database with a confidence score of 0.4 and visualized using R. Kinase activities were estimated using the KSEA algorithm (Casado et al., 2013) and the R implementation of KSEA App (Wiredja et al., 2017). Gene Ontology (GO) enrichment analysis was done using ViSEAGO package (Brionne et al., 2019) and p-values were assessed by a Fisher exact test. Kinase-substrate annotations were obtained from PhosphoSitePlus (PSP) (Wiredja et al., 2017) and the NetworKIN database (Linding et al., 2008). The analysis was performed with a minimum NetworKIN score of 5 for upregulated and downregulated phosphorylation sites with a p-value ≤ 0.05. Phosphosite-specific signature analysis was performed using PTMSEA (Krug et al., 2019). As input data p-values generated during statistical analysis were transformed and multiplied by the sign of the averaged log_2_-transformed fold changes. As identifier for the phosphorylation sites the flanking sequence (+/− 7 amino acids) was used. PTMSEA was done in R and PTMsigDB was used as reference dataset. Minimum overlap between PTM set and PTMsigDB was set to 10. Motif visualization of phospho-peptide sequences was done using ggseqlogo R package (Wagih, 2017). Prediction of protein disorder was done using the R implementation of the IUPred2A prediction tool (Mészáros et al., 2018). PPM1D-dependent/independent and etoposide-regulated phosphorylation sites were annotated with the phosphosite age feature from funscoR dataset (Ochoa et al., 2020). To assess statistical significance, Cochran-Armitage trend test was used and p-values were doubled to obtain two-sided alternative (p-value 0.00077). Multiple sequence alignment of KDT protein regions was done using Clustal Omega (Madeira et al., 2019).

## Data Availability

• The mass spectrometry-based proteomics data have been deposited to the ProteomeXchange Consortium via the PRIDE partner repository with the dataset identifier ProteomeXchange: PXD035420. • All original code is available in this paper’s supplemental information. • Any additional information required to reanalyze the data reported in this paper is available from the lead contact upon request.

## Supplemental information

Document S1. Figures S1–S6

Table S1. Phosphorylation sites quantified from U2OS cells after treatment with etoposide and PPM1Di, related to Figure 1

Table S2. Phosphorylation sites on proteins annotated with the GO terms RNA binding and DNA repair highlighted in Figure 1B, related to Figure 1

Table S3. Etoposide-induced and PPM1D-dependent phosphorylation sites with sequence motifs, related to Figure 2

Table S4. Phosphorylation sites quantified from HCT116 cells after treatment with etoposide and PPM1Di, related to Figure 5
