# Supplementary material for: Substrate spectrum of PPM1D in the cellular response to DNA double-strand breaks
Source: iScience. 2022 Aug 9;25(9):104892. doi: 10.1016/j.isci.2022.104892 (PMC9436757; doi:10.1016/j.isci.2022.104892)
Supplement: Document S1. Figures S1–S6 [file mmc1.pdf]

iScience, Volume *25*

Supplemental information

Substrate spectrum of PPM1D in the cellular response to DNA double-strand breaks

Justus F. Gräf, Ivan Mikicic, Xiaofei Ping, Claudia Scalera, Katharina Mayr, Lukas S. Stelzl, Petra Beli, and Sebastian A. Wagner

Figure S1

A

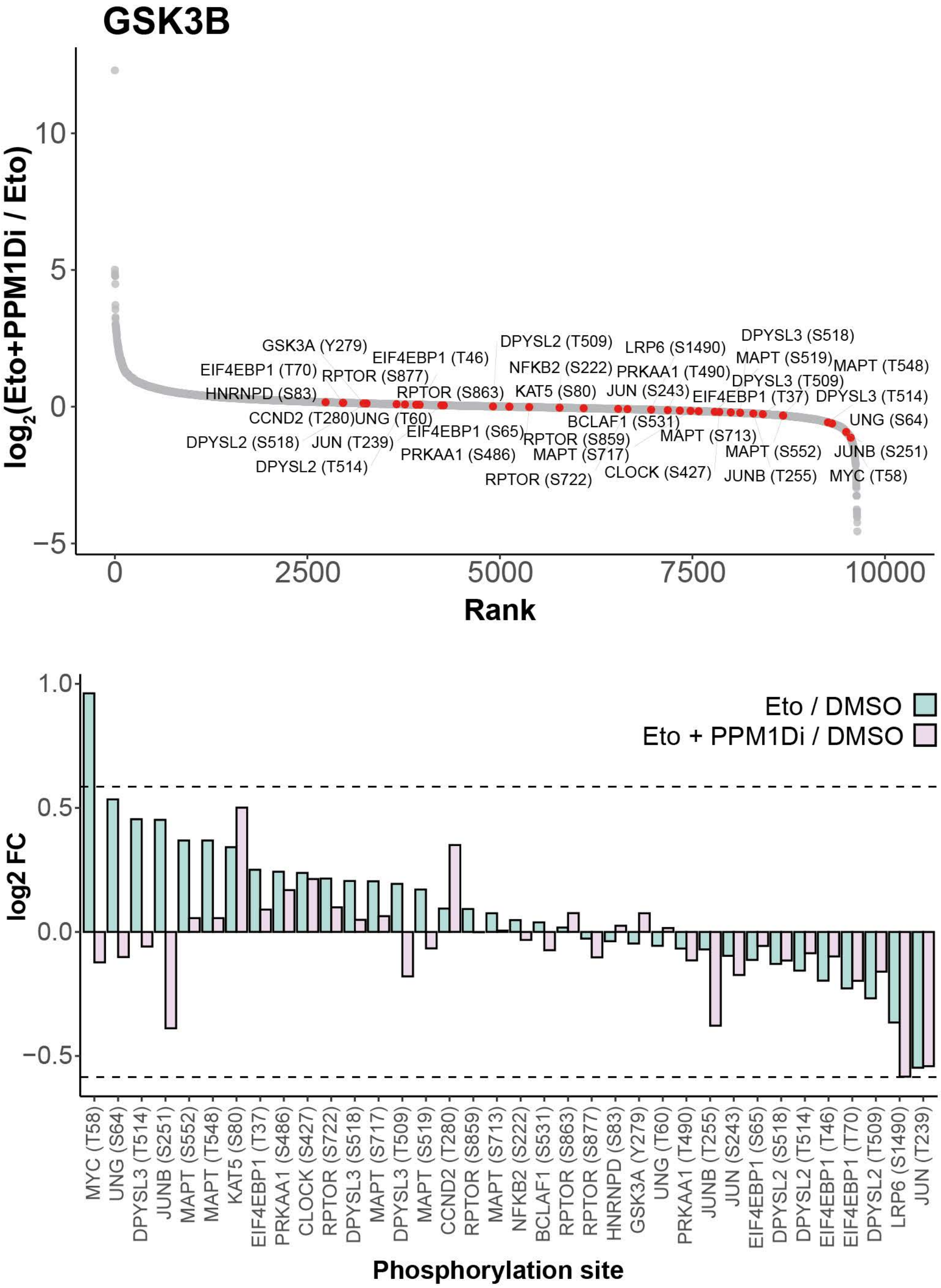
GSK3B
log2(Eto+PPM1Di / Eto)
10
5
0
−5
0
2500
5000
7500
10000
Rank
GSK3A (Y279)
EIF4EBP1 (T70)
HNRNPD (S83)
RPTOR (S877)
EIF4EBP1 (T46)
RPTOR (S863)
DPYSL2 (T509)
NFKB2 (S222)
KAT5 (S80)
LRP6 (S1490)
PRKAA1 (T490)
JUN (S243)
DPYSL3 (S518)
MAPT (S519)
DPYSL3 (T509)
MAPT (T548)
EIF4EBP1 (T37)
DPYSL3 (T514)
UNG (S64)
CCND2 (T280)
UNG (T60)
DPYSL2 (S518)
JUN (T239)
DPYSL2 (T514)
EIF4EBP1 (S65)
PRKAA1 (S486)
RPTOR (S859)
MAPT (S717)
RPTOR (S722)
BCLAF1 (S531)
CLOCK (S427)
MAPT (S713)
MAPT (S552)
JUNB (T255)
JUNB (S251)
MYC (T58)
1.0
0.5
0.0
−0.5
log2 FC
Eto / DMSO
Eto + PPM1Di / DMSO
MYC (T58)
UNG (S64)
DPYSL3 (T514)
JUNB (S251)
MAPT (S552)
MAPT (T548)
KAT5 (S80)
EIF4EBP1 (T37)
PRKAA1 (S486)
CLOCK (S427)
RPTOR (S722)
DPYSL3 (S518)
MAPT (S717)
DPYSL3 (T509)
MAPT (S519)
CCND2 (T280)
RPTOR (S859)
MAPT (S713)
NFKB2 (S222)
BCLAF1 (S531)
RPTOR (S863)
RPTOR (S877)
HNRNPD (S83)
GSK3A (Y279)
UNG (T60)
PRKAA1 (T490)
JUNB (T255)
JUN (S243)
EIF4EBP1 (S65)
DPYSL2 (S518)
DPYSL2 (T514)
EIF4EBP1 (T46)
EIF4EBP1 (T70)
DPYSL2 (T509)
LRP6 (S1490)
JUN (T239)
Phosphorylation site

Figure S1. PPM1Di treatment changes the phosphorylation landscape of GSK3B substrates, Related to Figure 1.

A) Phosphorylation sites ranked by FC after etoposide + PPM1Di in comparison to etoposide. PTMSEA-extracted GSK3B substrates are highlighted in red in the rank plot (top) and log_2-FCs for Eto/DMSO and Eto+PPM1Di/DMSO are plotted as bars (bottom).

Figure S2

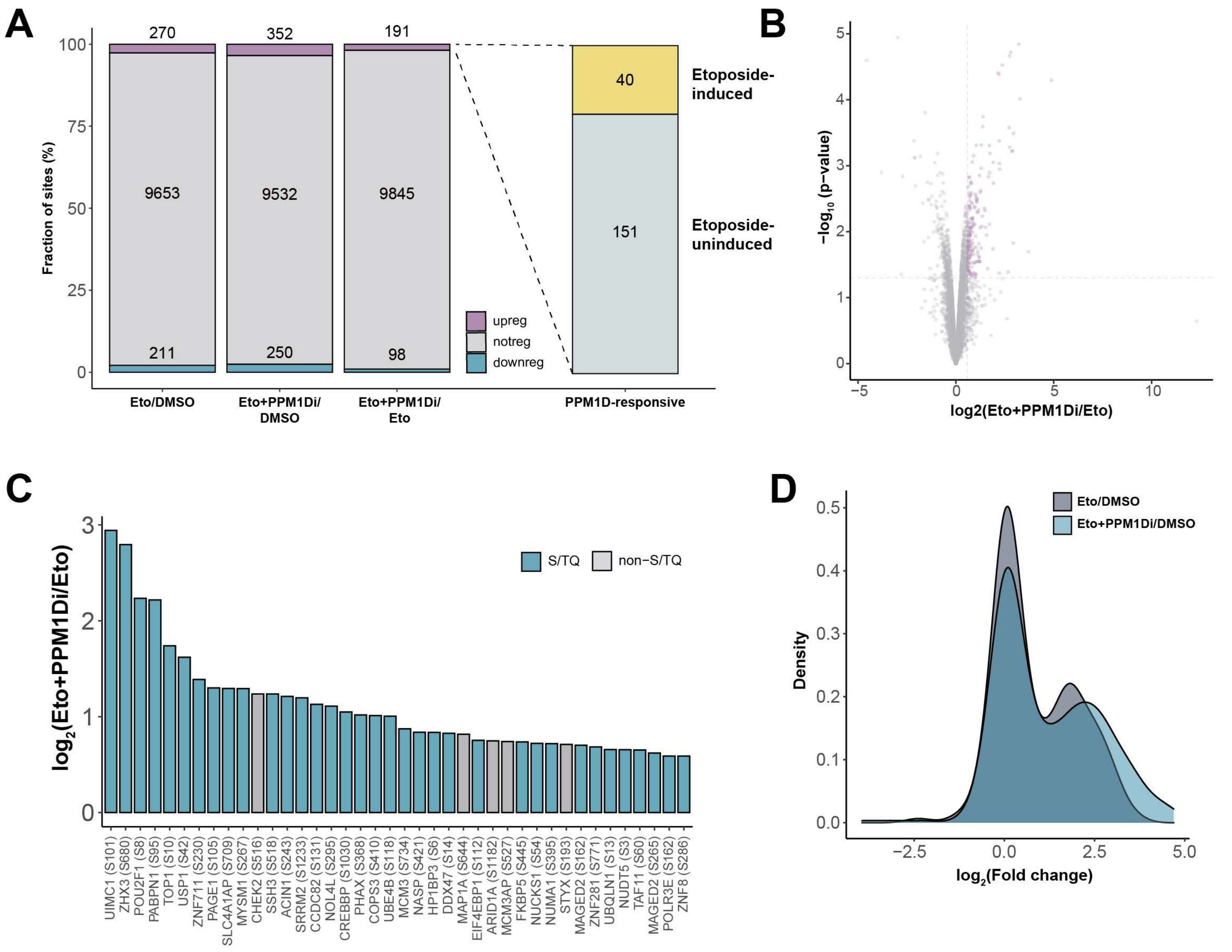
A
270
352
191
9653
9532
9845
211
250
98
40
151
Etoposide-induced
Etoposide-uninduced
Fraction of sites (%)
upreg
notreg
downreg
Eto/DMSO
Eto+PPM1Di/DMSO
Eto+PPM1Di/Eto
PPM1D-responsive
B
−log10 (p−value)
log2(Eto+PPM1Di/Eto)
C
log2(Eto+PPM1Di/Eto)
S/TQ
non−S/TQ
UIMC1 (S101)
ZHX3 (S680)
POU2F1 (S8)
PABPN1 (S95)
TOP1 (S10)
USP1 (S42)
ZNF711 (S230)
PAGE1 (S105)
SLC4A1AP (S709)
MYSM1 (S267)
CHEK2 (S516)
SSH3 (S518)
ACIN1 (S243)
SRRM2 (S1233)
CCDC82 (S131)
NOL4L (S295)
CREBBP (S1030)
PHAX (S368)
COPS3 (S410)
UBE4B (S118)
MCM3 (S734)
NASP (S421)
HP1BP3 (S6)
DDX47 (S14)
MAP1A (S644)
EIF4EBP1 (S112)
ARID1A (S1182)
MCM3AP (S527)
FKBP5 (S445)
NUCKS1 (S54)
NUMA1 (S395)
STYX (S193)
MAGED2 (S162)
ZNF281 (S771)
UBQLN1 (S13)
NUDT5 (S3)
TAF11 (S60)
MAGED2 (S265)
POLR3E (S162)
ZNF8 (S286)
D
Eto/DMSO
Eto+PPM1Di/DMSO
Density
log2(Fold change)

Figure S2. Quantitative phosphoproteomics identifies etoposide-dependent PPM1D substrates, Related to Figure 2.

A) Quantitative analysis of up- and downregulated phosphorylation sites after etoposide treatment and PPM1D inhibition (FC > 1.5, moderate t-test: p-value < 0.05). Upregulated sites after combined etoposide and PPM1Di treatment against etoposide-only condition are considered as PPM1D-responsive sites.

B) Volcano plot of phosphorylation sites after combined etoposide and PPM1Di treatment compared to etoposide treatment (FC > 1.5, moderate t-test: p-value < 0.05).

C) Barplot showing log_2-transformed FCs (Eto+PPM1D/Eto) of the 40 etoposide-induced and PPM1D-responsive phosphorylation sites. Sites with an S/TQ motif are annotated in blue.

D) Log_2-transformed FC densities of all phosphorylation sites with an S/TQ motif after etoposide treatment alone or in combination with PPM1D inhibition.

Figure S3

A

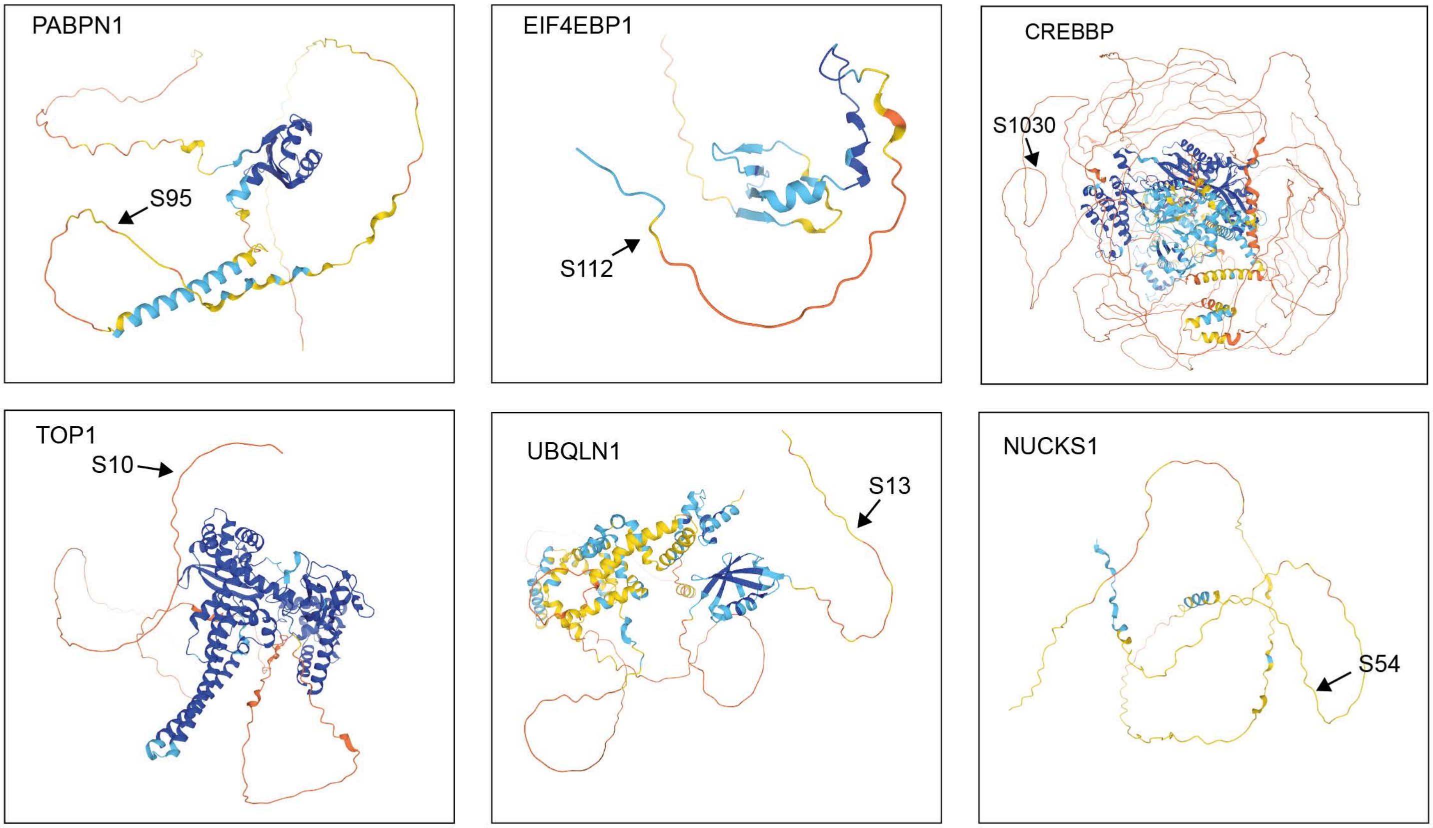

PABPN1
S95
EIF4EBP1
S112
CREBBP
S1030
TOP1
S10
UBQLN1
S13
NUCKS1
S54

B

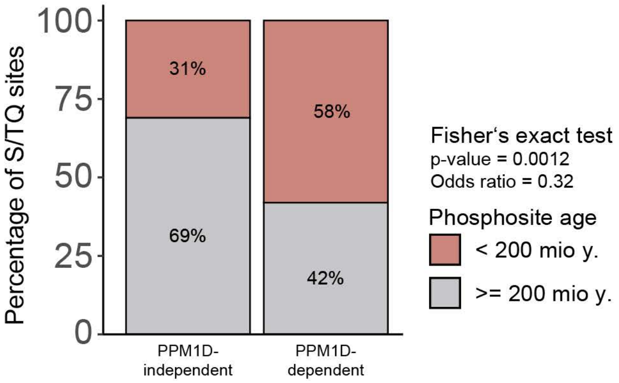

Percentage of S/TQ sites
100
75
50
25
0
31%
69%
58%
42%
PPM1D-independent
PPM1D-dependent
Fisher's exact test
p-value = 0.0012
Odds ratio = 0.32
Phosphosite age
< 200 mio y.
>= 200 mio y.

Figure S3. Multiple PPM1D-dependent phospho-sites localize to regions with high predicted intrinsic disorder, Related to Figure 4.

A) AlphaFold-predicted protein structures of selected PPM1D substrates with indicated localization of PPM1D-dependent phospho-sites.

B) Barplot showing fraction of S/TQ sites with phosphosite age < 200 mio years within PPM1D-dependent sites (upregulated in H/M condition) and PPM1D-independent sites (not upregulated in H/M condition) regardless of their regulation status after etoposide treatment. Fisher’s exact was carried out on the contingence table of S/TQ site counts in each subset.

Figure S4

A

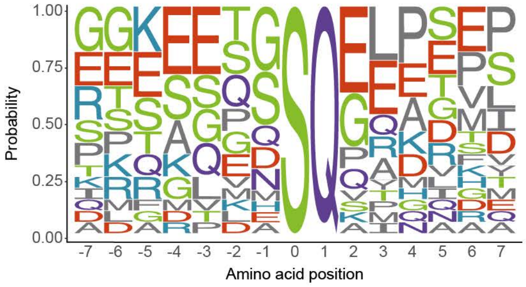

B

Phosphosite	Sequence motif
ZMYND11 (S421)	PKKEEPEPETEAVSSSQEIPTMPQPIEKVSV
TYMP (S23)	GTGAPPAPGDFSGEGSQGLPDPSPEPKQLPE
OXSR1 (S427)	AEPAKTAQALSSGSGSQETKIPISLVLRLRN
TCOF1 (S1410)	ASGDVKEKKGKGSLGSQGAKDEPEEELQKGM
SF3B2 (S289)	EKILQLKESRQEEMNSQQEEEEMETDARSSL
SMC1A (S957)	LSKGTMDDISQEEGSSQGEDSVSGSQRISSI
SMC1A (S358)	VEKARQEFEERMEEESQSQGRDLTLEENQVK
SMC1A (S360)	KARQEFEERMEEESQSQGRDLTLEENQVKKY
PPM1G (S183)	PPHSKSGGGTGEEPGSQGLNGEAGPEDSTRE
POLR1A (S1489)	PSLPALLTQPRKPTHSQEPQGPEAMERRVQA
H2AFX (S140)	VGPKAPSGGKKATQASQEY____________
HMGA1 (S44)	PRKQPPVSPGTALVGSQKEPSEVPTPKRPRG
HMGA1 (S9)	_______MSESSSKSSQPLASKQEKDGTEKR
BRD2 (S334)	ESGRPIKPPRKDLPDSQQQHQSSKKGKLSEQ
XPC (S351)	KERLTADPGGSSETSSQVLENHTKPKTSKGT
XPC (S892)	DAGGGLSSDEEEGTSSQAEAARILAASWPQN
MDC1 (S1086)	LPSIKPTVRKTRQDGSQEAPEAPLSSELEPF
PPP1R7 (S12)	____MAAERGAGQQQSQEMMEVDRRVESEES
MAPK14 (S2)	_______________MSQERPTFYRQELNKTI
METTL16 (S419)	QALEEKKPTPKESGNSQELARGPQERTPCGP
PNN (S381)	MEEETEVRESEKQQDSQPEEVMDVLEMVENV

Mean IUPred2A score = 0.81
Fraction with IUPred2a score > 0.5 = 95%

C

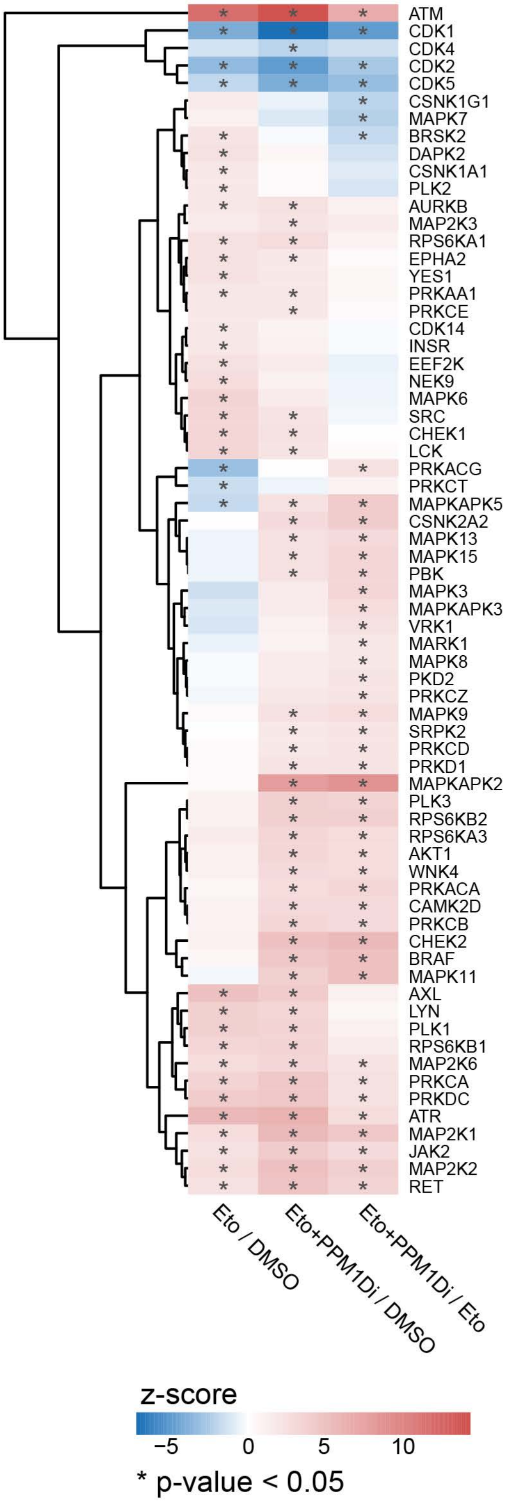

D

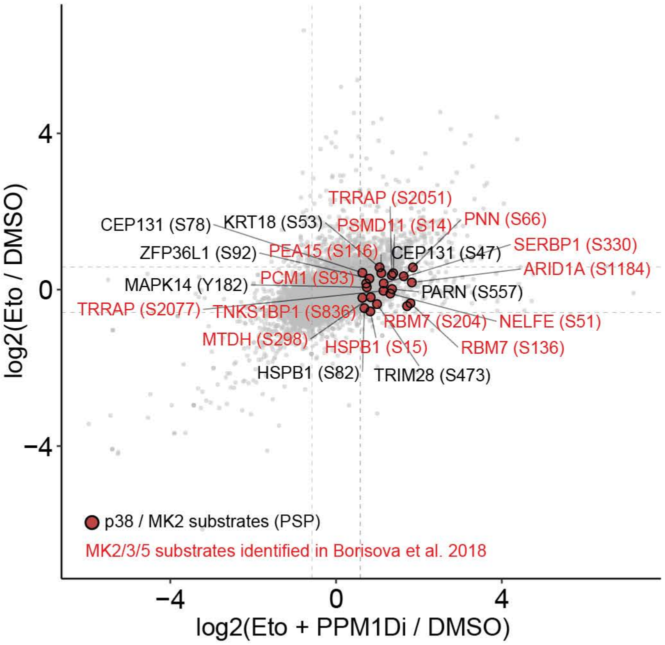

Figure S4. Phosphoproteomic analysis of PPM1D substrates after etoposide treatment in HCT116 cells, Related to Figure 5.

A) Sequence motif of etoposide-induced and PPM1D-dependent phosphosites identified in HCT116 cells but not in U2OS cells.

B) Phosphorylation site information and corresponding motif for each phosphosite corresponding to B).

C) Kinase-substrate enrichment analysis of phosphorylation sites from HCT116 screen. Relative z-score indicates changes in kinase activities after indicated treatments (P-values < 0.05 in one-tailed probability test are indicated with *).

D) Scatter plot showing log2 fold changes of Eto+PPM1Di/DMSO condition vs. Eto/DMSO condition. Highlighted points are p38 (MAPK14) and MK2 substrates (PSP) and labelled in red are MK2 substrates that were identified in Borisova et al. 2018.

Figure S5

A

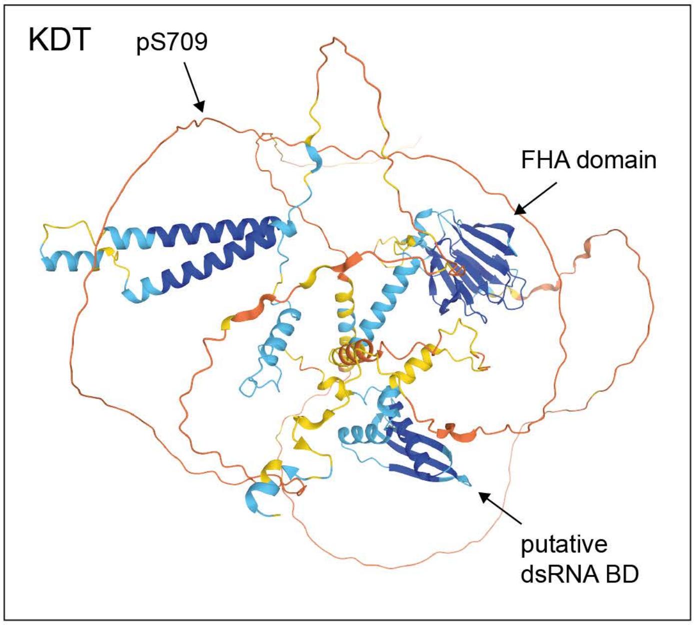

Figure S5. S709 of Kanadaptin localizes to a region with high predicted intrinsic disorder, Related to Figure 6.

A) AlphaFold-predicted structure of Kanadaptin with annotated FHA domain and dsRNA binding domain.

Figure S6

A

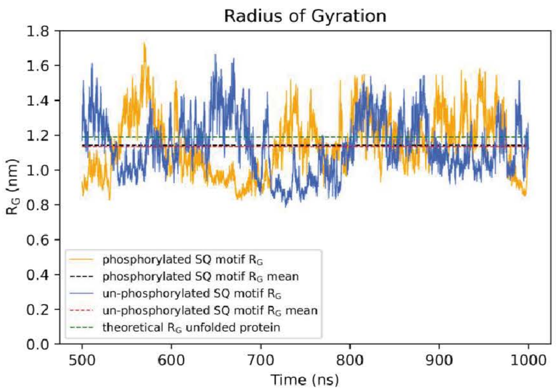

B

Secondary-structure un-phosphorylated SQ motif

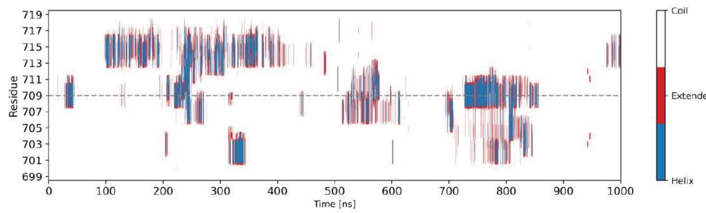

D

Solvent accessible surface area of residues (nm^2)

	Ser709	Glu713	Glu714	Glu714
un-phosphorylated SQ motif	0.84	1.45	1.48	1.34
phosphorylated SQ motif	1.68	1.47	1.53	1.39

C

Secondary-structure phosphorylated SQ motif

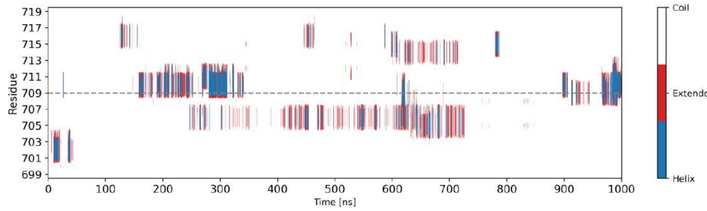

E

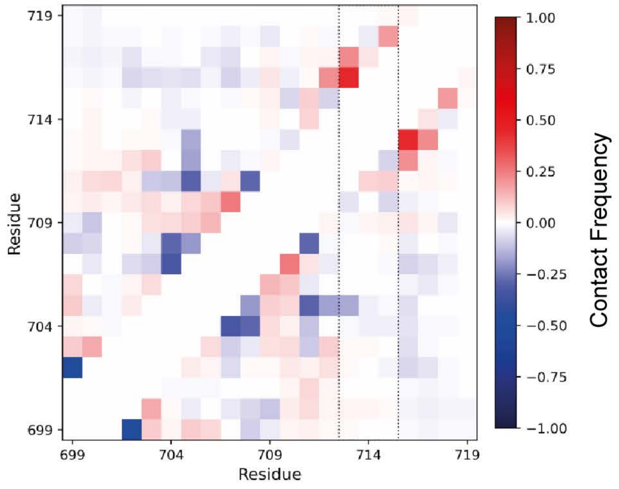

F

Un-phosphorylated SQ motif

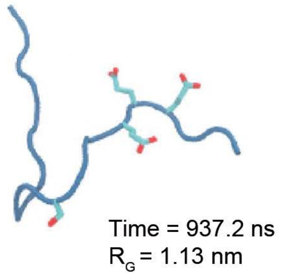

G

Phosphorylated SQ motif

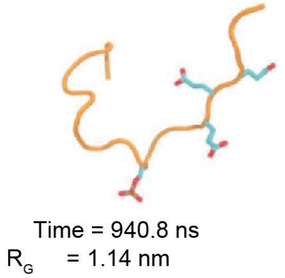

Figure S6. Characterization of the conformational dynamics of (un)phosphorylated Kanadaptin SQ motif, Related to Figure 7.

A) Extension of unphosphorylated and phosphorylated SQ motifs as tracked by radius of gyration (R_G).

B) and C) Secondary structure analysis of (un)phosphorylated SQ motifs using DSSP[1]. Overall, coils dominate as expected based on the disorder predictions. Close to the central serine and the triple glutamate motif, there is some capacity to form short and transient helical-like structures.

D) Solvent accessible surface area of S709 surrounding residues (nm^2) with (un)phosphorylated SQ motif.

E) Contact difference in simulation of unphosphorylated and phosphorylated SQ motifs. Contact map shows the difference in contact frequency between pairs of residues of unphosphorylated and phosphorylated SQ motifs in the MD simulation trajectories. Contact frequency differences were calculated by subtracting the contact frequency in the unphosphorylated SQ motif from the frequency in the phosphorylated SQ motif. Positive values (red) indicate pairs of residues that form more contacts in the simulation with the unphosphorylated SQ motif and blue contacts that are populated in the simulation of the phosphorylated SQ motif. Triple glutamate motif is highlighted with dashed lines.

F) and G) Structures with the average R_G from the simulations of (un)phosphorylated SQ motif. The R_G values of these structures are close to the average R_G values in the simulation and thus give an indication of the typical extension of the motif in the simulations. Central serine residue in the SQ motif is solvent-exposed as are the side chains of the triple glutamate motif.

Data S1. Source code employed for statistical analysis of the quantitative mass spectrometry data, Related to Figure 1, 2 and 5.

Substrate spectrum of PPM1D in the cellular response to DNA double strand breaks

Justus F. Gräf, Ivan Mikicic, Xiaofei Ping, Claudia Scalera, Katharina Mayr, Lukas S. Stelzl, Petra Beli, Sebastian A. Wagner

Statistical analysis of Etoposide + PPM1Di phosphoproteomic screens in U2OS and HCT116

SILAC setup:
H/L (Heavy / Light): Etoposide + PPM1Di / DMSO
H/M (Heavy / Medium): Etoposide + PPM1Di / Etoposide
M/L (Medium / Light): Etoposide / DMSO

```
############ Load and filter raw data

sites <- read.delim("Phospho (STY)Sites.txt")

sites <- filter(sites, Reverse != "+" &
                Potential.contaminant != "+" &
                Localization.prob >= 0.75)

sites$Position_first <- gsub("^(.*?)\\;.*$", "\\1", sites$Positions.within.proteins)
sites$names <- gsub("^(.*?)\\;.*$", "\\1", sites$Gene.names)
sites$names_site <- paste0(sites$names, " (", sites$Amino.acid, sites$Position_first, ")")
sites$Sequence.window.first <- gsub("^(.*?)\\;.*$", "\\1", sites$Sequence.window)
```

```
############ log2 transform MQ normalized H/L ratios
RatioCols_hl <- c("Ratio.H.L.normalized.1", "Ratio.H.L.normalized.2", "Ratio.H.L.normalized.3")

sites <-
  cbind(sites,
        log2=log2(sites[,RatioCols_hl]))


############ Build limma function for statistical analysis

significance_limma = function(ratios, max_q = 0.05, min_count = 2, prefix = '') {
  result = data.frame(
    count = ncol(ratios) - rowSums(is.na(ratios)),
    mean = rowMeans(ratios, na.rm = T)
  )
  fit = as.data.frame(eBayes(lmFit(ratios[result$count >= min_count, ])))
  result[result$count >= min_count, 'p'] = fit$p.value
  result[result$count >= min_count, 'q'] = p.adjust(fit$p.value, method = 'fdr')
  results = mutate(
    result,
    up = count >= min_count & mean > 1.5 & q <= max_q,
    down = count >= min_count & mean < 1/1.5 & q <= max_q

  )
  names(results) = paste0(prefix, names(results))
  return(results)
}


############ Run limma function and bind results to dataset

hl_statistics = significance_limma(
  log2(sites[,RatioCols_hl]))
colnames(hl_statistics) = paste0('Ratio.H.L.normalized.limma.', colnames(hl_statistics))
sites = bind_cols(sites, hl_statistics)


############ define cutoffs and assign regulation status
x1 = 1.5
y1 = 0.05

upreg_hl <- sites[,"Ratio.H.L.normalized.limma.mean"] > log2(x1) & sites[,"Ratio.H.L.normalized.limma.p"] < y1
downreg_hl <- sites[,"Ratio.H.L.normalized.limma.mean"] < -log2(x1) & sites[,"Ratio.H.L.normalized.limma.p"] < y1
notreg_hl <- !upreg_hl & !downreg_hl
```

```
############ log2 transform MQ normalized H/M ratios

RatioCols_hm <- c( "Ratio.H.M.normalized.1", "Ratio.H.M.normalized.2", "Ratio.H.M.normalized.3")

sites <-
  cbind(sites,
        log2=log2(sites[,RatioCols_hm]))


############ Build limma function for statistical analysis

significance_limma = function(ratios, max_q = 0.05, min_count = 2, prefix = '') {
  result = data.frame(
    count = ncol(ratios) - rowSums(is.na(ratios)),
    mean = rowMeans(ratios, na.rm = T)
  )
  fit = as.data.frame(eBayes(lmFit(ratios[result$count >= min_count, ])))
  result[result$count >= min_count, 'p'] = fit$p.value
  result[result$count >= min_count, 'q'] = p.adjust(fit$p.value, method = 'fdr')
  results = mutate(
    result,
    up = count >= min_count & mean > 1.5 & q <= max_q,
    down = count >= min_count & mean < 1/1.5 & q <= max_q
  )
  names(results) = paste0(prefix, names(results))
  return(results)
}


############ Run limma function and bind results to dataset

hm_statistics = significance_limma(
  log2(sites[,RatioCols_hm]))
colnames(hm_statistics) = paste0('Ratio.H.M.normalized.limma.', colnames(hm_statistics))
sites = bind_cols(sites, hm_statistics)


############ define cutoffs and assign regulation status

x1 = 1.5
y1 = 0.05

upreg_hm <- sites[,"Ratio.H.M.normalized.limma.mean"] > log2(x1) & sites[,"Ratio.H.M.normalized.limma.p"] < y1
downreg_hm <- sites[,"Ratio.H.M.normalized.limma.mean"] < -log2(x1) & sites[,"Ratio.H.M.normalized.limma.p"] < y1
notreg_hm <- !upreg_hm & !downreg_hm
```

```
############ log2 transform MQ normalized M/L ratios

RatioCols_ml <- c( "Ratio.M.L.normalized.1", "Ratio.M.L.normalized.2", "Ratio.M.L.normalized.3")

sites <-
  cbind(sites,
        log2=log2(sites[,RatioCols_ml]))

############ Build limma function for statistical analysis

significance_limma = function(ratios, max_q = 0.05, min_count = 2, prefix = '') {
  result = data.frame(
    count = ncol(ratios) - rowSums(is.na(ratios)),
    mean = rowMeans(ratios, na.rm = T)
  )
  fit = as.data.frame(eBayes(lmFit(ratios[result$count >= min_count, ])))
  result[result$count >= min_count, 'p'] = fit$p.value
  result[result$count >= min_count, 'q'] = p.adjust(fit$p.value, method = 'fdr')
  results = mutate(
    result,
    up = count >= min_count & mean > 1.5 & q <= max_q,
    down = count >= min_count & mean < 1/1.5 & q <= max_q
  )
  names(results) = paste0(prefix, names(results))
  return(results)
}


############ Run limma function and bind results to dataset

ml_statistics = significance_limma(
  log2(sites[,RatioCols_ml]))
colnames(ml_statistics) = paste0('Ratio.M.L.normalized.limma.', colnames(ml_statistics))
sites = bind_cols(sites, ml_statistics)


############ define cutoffs and assign regulation status

x1 = 1.5
y1 = 0.05

upreg_ml <- sites[,"Ratio.M.L.normalized.limma.mean"] > log2(x1) & sites[,"Ratio.M.L.normalized.limma.p"] < y1
downreg_ml <- sites[,"Ratio.M.L.normalized.limma.mean"] < -log2(x1) & sites[,"Ratio.M.L.normalized.limma.p"] < y1
notreg_ml <- !upreg_ml & !downreg_ml
```

Gene-Ontology enrichment analysis using ViSEAGO

The `go_analysis` function was run on gene names (selection) of regulated phosphosites of the corresponding condition indicated in figure descriptions against all other phosphosites in the dataset (background).

```
# background = background dataset (List of gene symbols as vector)
# selection = selection dataset (List of gene symbols as vector)
# ont_type = Ontology type (BP, MF or CC)


go_analysis <- function(background, selection, ont_type) {


  #UniprotDb <- read.table("data/Uniprot_db_2020_12_07.txt", header = TRUE, sep="\t")

  ############ Load EntrezGene db
  goterms <- read.table("data/gene2go.txt", sep="\t", header=T)

  EntrezGene <- newEntrezGene2Go()

  #check with this table for organism code (hs = 9606)
  #ViSEAGO::available_organisms(UniProt)

  myGENE2GO<-ViSEAGO::annotate("9606", EntrezGene)


  ############ Annotate data with EntrezID

  symbols_sel <- selection
  cols <- c("SYMBOL", "ENTREZID")
  selection_df <- AnnotationDbi::select(org.Hs.eg.db, keys = symbols_sel, columns = cols, keytype = "SYMBOL")
  selection <- selection_df$ENTREZID
  names(selection) <- selection_df$SYMBOL

  symbols_back <- background
  cols <- c("SYMBOL", "ENTREZID")
  background_df <- AnnotationDbi::select(org.Hs.eg.db, keys=symbols_back, columns=cols, keytype="SYMBOL")
  background <- background_df$ENTREZID
  names(background) <- background_df$UNIPROT


  ############ Create topGO data
  GO <- create_topGOdata(
    geneSel=selection,
    allGenes=background,
    gene2GO=myGENE2GO,
    ont=ont_type,
    nodeSize=5)


  ############ Run fisher's exact test
  classic <- runTest(
    GO,
    algorithm ="classic",
    statistic = "fisher")

  ############ Combine topGO data and test results
  GO_Res <- merge_enrich_terms(
    Input=list(
      condition=c("GO", "classic")))



  GO_RESULTS <- read.table("tables/GO_RESULTS.txt", header=TRUE)
  GO_RESULTS <- GO_RESULTS[order(GO_RESULTS$condition.pvalue), ]


  GO_RESULTS$Enrichment <- gsub("^(.*?)\\%.*$", "\\1", GO_RESULTS$condition.genes_frequency)
  GO_RESULTS <- GO_RESULTS[, c(1, 2, 8, 3:7)]

  write.table(GO_RESULTS, "tables/GO_RESULTS.txt", row.names = F, sep="\t")

}
```

Kinase-substrate enrichment analysis (KSEA)

```
############ Load combined PSP and NetworKIN database

#KSPSP2020NetworKIN <- read.table("KSPSP2020NetworKIN.txt")
sites$Residue <- paste0(sites$Amino.acid, sites$Position)


############ Extract required phosphosite information from dataset

ksea_cn_a <- c("Protein", "names", "Sequence.window.first", "Residue", "Ratio.M.L.normalized.limma.p", "Ratio.M.L
.normalized.limma.mean")
df_a <- sites[, ksea_cn_a]
colnames(df_a) <- c("Protein", "Gene", "Peptide", "Residue.Both", "p", "FC")
df_a$FC <- 2^df_a$FC

df_a <- df_a[complete.cases(df_a), ]


############ Run KSEA.Complete function on selected data

KSEA.Complete(KSPSP2020NetworKIN, PX=df_a,
              NetworKIN = TRUE,
              NetworKIN.cutoff = 10,
              m.cutoff = 5,
              p.cutoff = 0.05
            )

file_name <- "KSEA Kinase Scores.csv"

KSEA <- read.delim(file.path( file_name),
                    stringsAsFactors=FALSE, header = TRUE, sep = ",")


############ Filter for cutoff

KSEA_cutoff <- transform(KSEA, cutoff = p.value <= 0.05)


upreg_a <- KSEA_cutoff[, "z.score"] > 0 &
  KSEA_cutoff[, "cutoff"] == TRUE

downreg_a <- KSEA_cutoff[, "z.score"] < 0 &
  KSEA_cutoff[, "cutoff"] == TRUE

notreg_a <- !upreg_a & !downreg_a

KSEA_cutoff_ML <- KSEA_cutoff
```

```
############ Extract required phosphosite information from dataset

ksea_cn_b <- c("Protein", "names", "Sequence.window.first", "Residue", "Ratio.H.L.normalized.limma.p", "Ratio.H.L
.normalized.limma.mean")
df_b <- sites[, ksea_cn_b]
colnames(df_b) <- c("Protein", "Gene", "Peptide", "Residue.Both", "p", "FC")
df_b$FC <- 2^df_b$FC

df_b <- df_b[complete.cases(df_b), ]


############ Run KSEA.Complete function on selected data


KSEA.Complete(KSPSP2020NetworKIN, PX=df_b,
              NetworKIN = TRUE,
              NetworKIN.cutoff = 10,
              m.cutoff = 5,
              p.cutoff = 0.05
            )

file_name <- "KSEA Kinase Scores.csv"


KSEA <- read.delim(file.path( file_name),
                    stringsAsFactors=FALSE, header = TRUE, sep = ",")


############ Filter for cutoff


KSEA_cutoff <- transform(KSEA, cutoff = p.value <= 0.05)


upreg_b <- KSEA_cutoff[, "z.score"] > 0 &
  KSEA_cutoff[, "cutoff"] == TRUE

downreg_b <- KSEA_cutoff[, "z.score"] < 0 &
  KSEA_cutoff[, "cutoff"] == TRUE

notreg_b <- !upreg_b & !downreg_b


KSEA_cutoff_HL <- KSEA_cutoff
```

```
############ Extract required phosphosite information from dataset

ksea_cn_c <- c("Protein", "names", "Sequence.window.first", "Residue", "Ratio.H.M.normalized.limma.p", "Ratio.H.M
.normalized.limma.mean")
df_c <- sites[, ksea_cn_c]
colnames(df_c) <- c("Protein", "Gene", "Peptide", "Residue.Both", "p", "FC")
df_c$FC <- 2^df_c$FC

df_c <- df_c[complete.cases(df_c), ]

############ Run KSEA.Complete function on selected data


KSEA.Complete(KSPSP2020NetworKIN, PX=df_c,
              NetworKIN = TRUE,
              NetworKIN.cutoff = 10,
              m.cutoff = 5,
              p.cutoff = 0.05
            )

file_name <- "KSEA Kinase Scores.csv"


KSEA <- read.delim(file.path( file_name),
                    stringsAsFactors=FALSE, header = TRUE, sep = ",")


############ Filter for cutoff

KSEA_cutoff <- transform(KSEA, cutoff = p.value <= 0.05)


upreg_c <- KSEA_cutoff[, "z.score"] > 0 &
  KSEA_cutoff[, "cutoff"] == TRUE

downreg_c <- KSEA_cutoff[, "z.score"] < 0 &
  KSEA_cutoff[, "cutoff"] == TRUE

notreg_c <- !upreg_c & !downreg_c

KSEA_cutoff_HM <- KSEA_cutoff
```

```
sessionInfo()
```

```
## R version 4.1.3 (2022-03-10)
## Platform: x86_64-apple-darwin17.0 (64-bit)
## Running under: macOS Big Sur/Monterey 10.16
##
## Matrix products: default
## BLAS:   /Library/Frameworks/R.framework/Versions/4.1/Resources/lib/libRblas.0.dylib
## LAPACK: /Library/Frameworks/R.framework/Versions/4.1/Resources/lib/libRlapack.dylib
##
## locale:
## [1] de_DE.UTF-8/de_DE.UTF-8/de_DE.UTF-8/C/de_DE.UTF-8/de_DE.UTF-8
##
## attached base packages:
## [1] stats     graphics  grDevices utils     datasets  methods   base
##
## other attached packages:
##  [1] ViSEAGO_1.8.0   limma_3.50.3    forcats_0.5.1   stringr_1.4.0
##  [5] dplyr_1.0.9     purrr_0.3.4     readr_2.1.2     tidyr_1.2.0
##  [9] tibble_3.1.7    ggplot2_3.3.6   tidyverse_1.3.1 KSEAapp_0.99.0
##
## loaded via a namespace (and not attached):
##   [1] fgsea_1.20.0           colorspace_2.0-3       ellipsis_0.3.2
##   [4] dynamicTreeCut_1.63-1  XVector_0.34.0         fs_1.5.2
##   [7] rstudioapi_0.13        topGO_2.46.0           DT_0.23
##  [10] bit64_4.0.5            AnnotationDbi_1.56.2   fansi_1.0.3
##  [13] lubridate_1.8.0        xml2_1.3.3             codetools_0.2-18
##  [16] R.methodsS3_1.8.1      cachem_1.0.6           GOSemSim_2.20.0
##  [19] knitr_1.39             jsonlite_1.8.0         broom_0.8.0
##  [22] GO.db_3.14.0           dbplyr_2.1.1           png_0.1-7
##  [25] R.oo_1.24.0            graph_1.72.0           DiagrammeR_1.0.9
##  [28] compiler_4.1.3         httr_1.4.3             backports_1.4.1
##  [31] lazyeval_0.2.2         Matrix_1.4-1           assertthat_0.2.1
##  [34] fastmap_1.1.0          cli_3.3.0              visNetwork_2.1.0
##  [37] htmltools_0.5.2        prettyunits_1.1.1      tools_4.1.3
##  [40] igraph_1.3.1           gtable_0.3.0           glue_1.6.2
##  [43] GenomeInfoDbData_1.2.7 rappdirs_0.3.3         fastmatch_1.1-3
##  [46] Rcpp_1.0.8.3           Biobase_2.54.0         cellranger_1.1.0
##  [49] jquerylib_0.1.4        vctrs_0.4.1            Biostrings_2.62.0
##  [52] iterators_1.0.14       xfun_0.31              rvest_1.0.2
##  [55] lifecycle_1.0.1        gtools_3.9.2.1         dendextend_1.15.2
##  [58] XML_3.99-0.9           zlibbioc_1.40.0        scales_1.2.0
##  [61] TSP_1.2-0              hms_1.1.1              parallel_4.1.3
##  [64] SparseM_1.81           RColorBrewer_1.1-3     yaml_2.3.5
##  [67] curl_4.3.2             heatmaply_1.3.0        memoise_2.0.1
##  [70] gridExtra_2.3          UpSetR_1.4.0           sass_0.4.1
##  [73] biomaRt_2.50.3         stringi_1.7.6          RSQLite_2.2.14
##  [76] S4Vectors_0.32.4       foreach_1.5.2          seriation_1.3.5
##  [79] filelock_1.0.2         caTools_1.18.2         BiocGenerics_0.40.0
##  [82] BiocParallel_1.28.3    GenomeInfoDb_1.30.1    rlang_1.0.2
##  [85] pkgconfig_2.0.3        bitops_1.0-7           matrixStats_0.62.0
##  [88] evaluate_0.15          lattice_0.20-45        htmlwidgets_1.5.4
##  [91] bit_4.0.4              tidyselect_1.1.2       AnnotationForge_1.36.0
##  [94] plyr_1.8.7             magrittr_2.0.3         R6_2.5.1
##  [97] IRanges_2.28.0         gplots_3.1.3           generics_0.1.2
## [100] DBI_1.1.2              pillar_1.7.0           haven_2.5.0
## [103] withr_2.5.0            KEGGREST_1.34.0        RCurl_1.98-1.6
## [106] modelr_0.1.8           crayon_1.5.1           KernSmooth_2.23-20
## [109] utf8_1.2.2             plotly_4.10.0          BiocFileCache_2.2.1
## [112] tzdb_0.3.0             rmarkdown_2.14         viridis_0.6.2
## [115] progress_1.2.2         grid_4.1.3             readxl_1.4.0
## [118] data.table_1.14.2      blob_1.2.3             webshot_0.5.3
## [121] reprex_2.0.1           digest_0.6.29          R.utils_2.11.0
## [124] stats4_4.1.3           munsell_0.5.0          registry_0.5-1
## [127] viridisLite_0.4.0      bslib_0.3.1
```
